# Renal subcapsular delivery of PGE_2_ promotes kidney repair by activating endogenous Sox9^+^ stem cells

**DOI:** 10.1016/j.isci.2021.103243

**Published:** 2021-10-08

**Authors:** Shang Chen, Haoyan Huang, Yue Liu, Chen Wang, Xiaoniao Chen, Yuqiao Chang, Yuhao Li, Zhikun Guo, Zhibo Han, Zhong-Chao Han, Qiang Zhao, Xiang-Mei Chen, Zongjin Li

**Affiliations:** 1 School of Medicine, Nankai University, 94 Weijin Road, Tianjin 300071, China; 2 The Key Laboratory of Bioactive Materials, Ministry of Education, Nankai University, The College of Life Sciences, Tianjin, China; 3 Beijing Tongren Eye Center, Beijing Tongren Hospital, Capital Medical University, Beijing, China; 4 Henan Key Laboratory of Medical Tissue Regeneration, Xinxiang Medical University, Xinxiang, China; 5 Jiangxi Engineering Research Center for Stem Cell, Shangrao, Jiangxi, China; 6 Tianjin Key Laboratory of Engineering Technologies for Cell Pharmaceutical, National Engineering Research Center for Cell Products, AmCellGene Co., Ltd., Tianjin China; 7 Beijing Engineering Laboratory of Perinatal Stem Cells, Beijing Institute of Health and Stem Cells, Health & Biotech Co., Beijing, China; 8 State Key Laboratory of Kidney Diseases, Chinese PLA General Hospital, 28 Fuxing Road, Beijing 100039, China

**Keywords:** Drug delivery system, Pathophysiology, Cell biology, Stem cells research

## Abstract

Prostaglandin E_2_ (PGE_2_) has recently been recognized to play a role in immune regulation and tissue regeneration. However, the short half-life of PGE_2_ limits its clinical application. Improving the delivery of PGE_2_ specifically to the target organ with a prolonged release method is highly desirable. Taking advantage of the adequate space and proximity of the renal parenchyma, renal subcapsular delivery allows minimally invasive and effective delivery to the entire kidney. Here, we report that by covalently cross-linking it to a collagen matrix, PGE_2_ exhibits an adequate long-term presence in the kidney with extensive intraparenchymal penetration through renal subcapsular delivery and significantly improves kidney function. Sox9 cell lineage tracing with intravital microscopy revealed that PGE_2_ could activate the endogenous renal progenitor Sox9^+^ cells through the Yap signaling pathway. Our results highlight the prospects of utilizing renal subcapsular-based drug delivery and facilitate new applications of PGE_2_-releasing matrices for regenerative therapy.

## Highlights

• PGE_2_ exhibits an adequate long-term release by being covalently cross-linked to collagen
• The renal subcapsular space serves as a reservoir for the delivery of PGE_2_
• Sox9^+^ renal progenitor cells can be lineage traced intravitally by microscopy
• PGE_2_ activates the endogenous renal progenitor Sox9^+^ cells through the YAP pathway

## Graphical abstract

**Figure:**
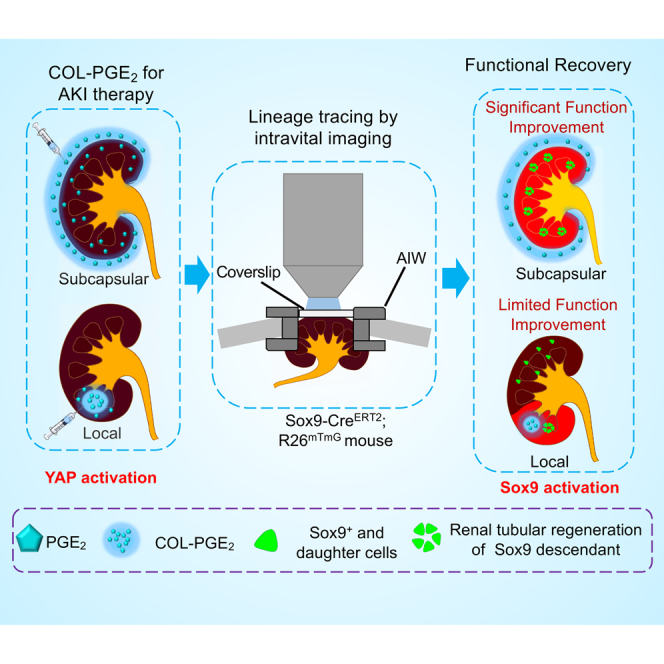

## Introduction

Acute kidney injury (AKI), which leads to abrupt loss of kidney function, is highly associated with the subsequent risks of chronic kidney disease and end-stage renal disease, which will lead to the need for lifelong dialysis and renal replacement therapy and cause high morbidity and mortality (James et al., 2020; Liu et al., 2020; Zhang et al., 2020; Zhang and Li, 2020). Methods for delivering stem cells, cytokines, and biomaterials to the kidney, such as systemic administration, intrarenal parenchyma injection, or intrarenal artery injection, offer promise for kidney regeneration after AKI (Fazekas and Griffin, 2020; Gao et al., 2020; Gonsalez et al., 2019; Little and Kairath, 2016). Targeting delivery specifically to the whole kidney, by which therapeutic drug levels can be maintained over time, is highly desirable. Local delivery results in high drug deposition and even toxic concentrations but leads to a dose that is lower than that required in more distant areas of the injected tissue. Intrarenal artery injection or systemic administration will also lead to poor local retention and insufficient distribution throughout the entire renal parenchyma over time (Segura-Ibarra et al., 2017). Renal subcapsular spaces under kidney capsules located on the surface of the kidneys have been widely used for cell or tissue transplantation because cells exhibit better growth in these spaces (Cunha and Baskin, 2016; van den Berg et al., 2018), and this provides insight into the use of subcapsular transplantation for AKI therapy to increase drug concentrations in the whole kidney (Dankers et al., 2015; Morizane and Bonventre, 2017; Segura-Ibarra et al., 2017).

Prostaglandin E_2_ (PGE_2_), a kind of prostaglandin, has been recognized to play an important role in tissue regeneration and maintenance when the concentration of PGE_2_ in injured tissues is elevated (Palla et al., 2021; Zhang et al., 2015). PGE_2_ is synthesized by cyclooxygenase (COX) and prostaglandin E synthases (PGES) from arachidonic acid and interacts with four G-protein-coupled E-prostanoid receptors designated EP1, EP2, EP3, and EP4 to cause various downstream effects, including angiogenesis, inflammation, and stem cell development (Cheng et al., 2021; Norregaard et al., 2015; Zhang and Daaka, 2011). Recent studies revealed that injury-induced PGE_2_ secretion is key to activating endogenous stem/progenitor cells for myocardial regeneration after infarction (FitzSimons et al., 2020; Hsueh et al., 2014). Moreover, PGE_2_ shows obvious anti-inflammatory and pro-angiogenesis effects by inducing macrophage polarization from the M1 phenotype to the M2 phenotype at injured sites. In addition, PGE_2_ in hepatocytes inhibits the activation of hepatic stellate cells and attenuates liver fibrosis in mice (Brea et al., 2018). Increasing evidence revealed that PGE_2_ is a promising therapeutic candidate for improving tissue repair and regeneration, reflected mainly in improving cutaneous wound healing, cardiac protection function, and accelerating liver regeneration (Chakkour and Kreydiyyeh, 2019; Xiao et al., 2004; Zhang et al., 2015, 2018). Furthermore, the main therapeutic mechanism of mesenchymal stem cells (MSCs) in tissue repair is mediated by the secretion of PGE_2_ by MSCs (Cao et al., 2020; Du et al., 2016; Fazekas and Griffin, 2020; Zhang et al., 2018). However, the short half-life of PGE_2_ *in vivo* limits its therapeutic efficiency in translational applications (Zhang et al., 2018). PGE_2_ has a faster turnover rate of approximately 30 s in circulation due to degradation by 15-hydroxyprostaglandin dehydrogenase (15-PGDH), converting it into an inactivated 15-keto-PGE_2,_ which limits its use to biomedical applications (Bygdeman, 2003; Kozak et al., 2001; Zhang et al., 2015). The strategy of conjugating PGE_2_ by binding to biomaterials will maintain the presence of PGE_2_ *in situ*, further enhancing the therapeutic effects of PGE_2_ (Yao et al., 2015; Zhao et al., 2019). By facilitating the controlled release of PGE_2_ *in situ*, renal subcapsular delivery provides a reservoir that allows the diffusion of PGE_2_ into the entire kidney. Subcapsular delivery through sustained release of therapeutic drugs could avoid injury to the renal parenchyma and can result in maximal therapeutic efficacy in the kidney and minimal systemic side effects (Dankers et al., 2015).

Currently, it is believed that the kidney can regenerate itself after injury through dedifferentiation of tubular epithelial cells in the nephron or activation of resident/endogenous stem cells in the kidney (Zhang et al., 2020; Zhang and Li, 2020). However, the mechanisms of renal regeneration after AKI with or without treatment are poorly understood. Lineage tracing and two-photon intravital microscopy methods offer an opportunity to visualize cell behavior and examine dynamic cellular processes at the single-cell level in the context of intact organisms instead of in the *in vitro* environment (Boulch et al., 2019; Eles et al., 2019; Eng et al., 2018; Fan et al., 2018; Kaverina et al., 2019). Lineage tracing of kidney cells in living animals with intravital microscopy will unveil the potency and activities of renal resident cells after AKI (Liu and Li, 2021; Zhang and Li, 2020) and help to assess and determine the optimal therapeutics for AKI treatment. Sex-determining region Y box 9 (Sox9) has been shown to be a marker of renal progenitor cells that contributes to renal regeneration after AKI (Kang et al., 2016; Zhang and Li, 2020). To date, the therapeutic effect of PGE_2_ through the promotion of Sox9^+^ cell proliferation in AKI has not been investigated.

In addition, Hippo/Yap (Yes-associated protein) signaling pathway is composed of a series of conservative kinases, which mainly control organ size by regulating cell proliferation and apoptosis (Zhou et al., 2020). Specifically, the Yap signal controls genes that regulate cell and organism metabolism and coordinate organ growth and homeostasis with nutrition and metabolism (Moya and Halder, 2019). Several studies have revealed that activating Yap favors the regeneration of organs with poor or damaged regenerative capacity, such as the heart of adult mice (Li et al., 2020), the liver and intestines of elderly or diseased mice (Kim et al., 2017; Loforese et al., 2017), and injured skin (Moya and Halder, 2019). Limited, but convincing, evidence suggests that Yap plays an essential role in kidney and urinary tract development, podocyte homeostasis, fibrosis, and cystic and diabetic nephropathy (Chen et al., 2018; Wong et al., 2016).

The objective of this study was to investigate the potential use of the renal subcapsular space as a reservoir for the delivery of PGE_2_ to the kidney. By covalently cross-linking it to a collagen matrix scaffold, we hypothesized that PGE_2_ could significantly improve AKI via a site-specific, controlled-release drug delivery system. Furthermore, intravital microscopy was used to examine the spatiotemporal kinetics of kidney progenitor Sox9^+^ cells under PGE_2_ treatment using inducible Sox9 transgenic mice. Our results highlight the prospect of utilizing the renal subcapsular space for drug delivery for the treatment of kidney diseases. Furthermore, our data suggested that PGE_2_ could promote renal regeneration by activating Sox9 through the Yap signaling pathway.

## Results

### Synthesis and characterization of the COL-PGE_2_ matrix

**Figure 1:**
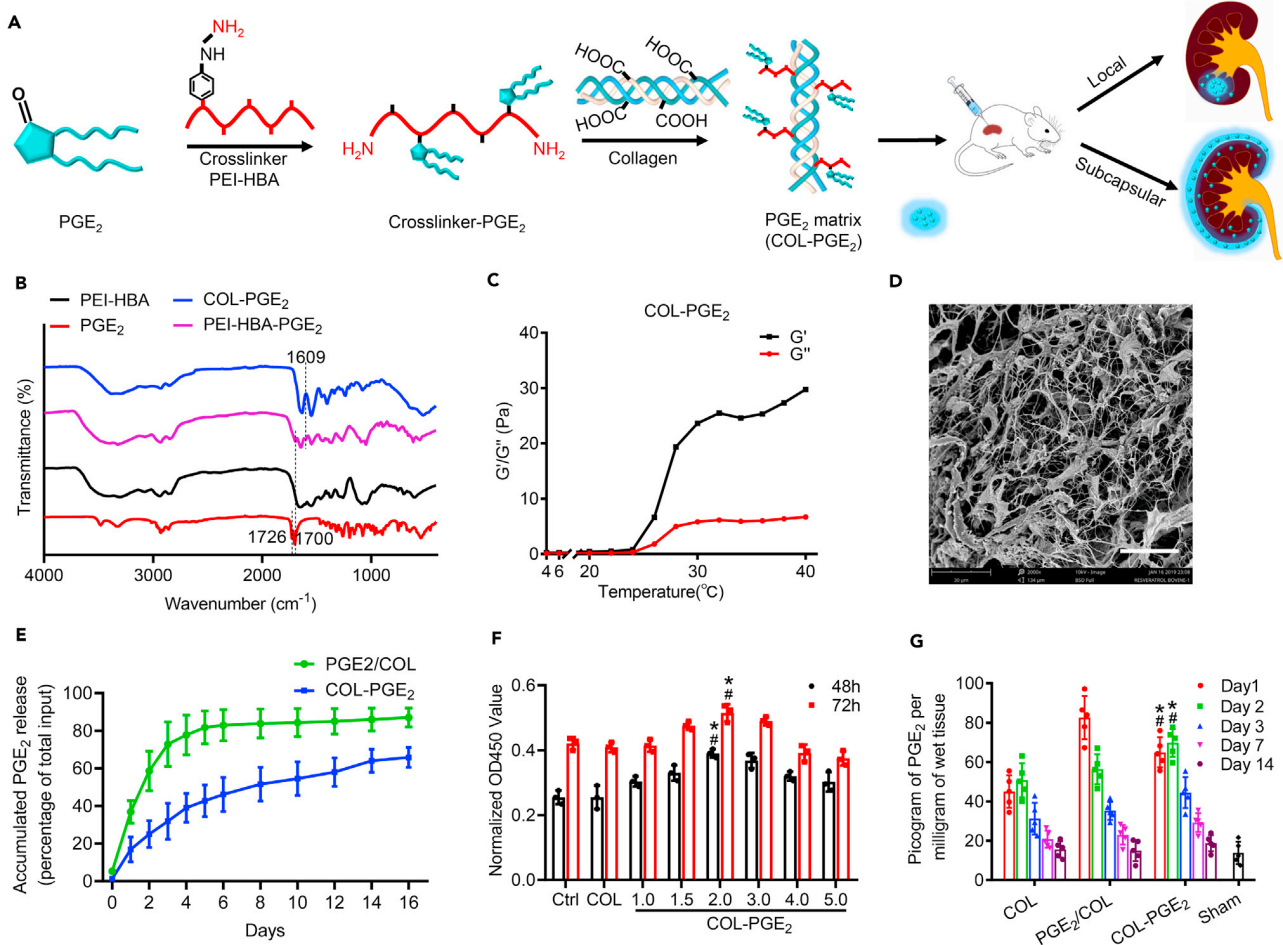
Preparation and characterization of the COL-PGE_2_ matrix (A) A schematic depicting the method of synthesis of the COL-PGE_2_matrix. (B) FT-IR analysis shows the characteristic peaks of the PGE_2_, PEI (polyethyleneimine)-HBA (4-hydrazinobenzoic acid), PEI-HBA-PGE_2_, and COL-PGE_2_ matrix. A peak at 1,609 cm^−1^ revealed the successful conjugation of PGE_2_. (C) Evaluation of the rheological profile of the COL-PGE_2_ matrix by analyzing the storage modulus (G′) and the loss modulus (G″) with temperature changes, which indicated the phase transition of the COL-PGE_2_ matrix from a solution to a gel. Pa, pascal (unit of pressure or stress). (D) Scanning electron micrograph image that reveals the morphologic structure of the lyophilized COL-PGE_2_ matrix. Scale bars, 30 μm. (E) *In vitro* release of PGE_2_ by the COL-PGE_2_ matrix. The COL-PGE_2_ matrix was deposited in a test tube and covered with a layer of 1× PBS buffer. The amount of released PGE_2_ was determined by ELISA. Two-way repeated measures ANOVA with Sidak’s post hoc test was used for statistical analysis. Data are expressed as mean ± SD; n = 3. The mixture of free PGE_2_ with collagen was tested as a control, and the differences were significant between the COL-PGE_2_ matrix (COL-PGE_2_) and the physical mix of free PGE_2_ physical mix with collagen type I (PGE_2_/COL) from day 1 to day 16. (F) CCK-8 assay showing proliferation of primary renal tubular epithelial cells with different concentrations of the COL-PGE_2_ matrix. Two-way multivariate analysis of variance (two-way MANOVA) with the Tukey post hoc test was used for statistical analysis. Data are expressed as mean ± SD; n = 3, ∗p < 0.05 versus COL; ^#^p < 0.05 versus control. (G) For *in vivo* PGE_2_ release, COL, PGE_2_/COL, and COL-PGE_2_ matrices were injected into the renal capsule of C57BL/6 mice after AKI. The kidney was then explanted by removing the kidney capsule and matrix at the indicated time points for the assessment of the released PGE_2_ by ELISA. The concentration of PGE_2_ in the kidney remains at high level from day 1 to day 3 and lasts for more than two weeks in the COL-PGE_2_ group. Furthermore, PGE_2_ can be found in normal tissue (Sham group), and injury (COL group) can stimulate PGE_2_ secretion, but PGE_2_ is at a low level. Two-way MANOVA with the Tukey post hoc test was used for statistical analysis. Data are expressed as mean ± SD; n = 5, ∗p < 0.05 versus COL; ^#^p < 0.05 versus PGE_2_/COL.

PGE_2_ is rapidly metabolized *in vivo,* and the half-life of PGE_2_ in the circulatory system is approximately 30 s (Bygdeman, 2003; Kozak et al., 2001; Zhang et al., 2018). To improve the therapeutic efficacy of PGE_2_, we developed a PGE_2_ release matrix by cross-linking collagen type I with 4-hydrazinobenzoic acid (HBA)-polyethyleneimine (PEI), which was achieved by the reaction of the amine group on HBA with the carbonyl group on PGE_2_ through condensation reactions (Figures 1A and S1A–S1C). The successful conjugation of HBA to PEI was confirmed by the appearance of carbonyl (C=O, ∼1,651 cm^−1^) and N–N bond (∼1,551 cm^−1^) vibrations in the Fourier transform infrared (FT-IR) spectroscopic analysis (Figure S2A). FT-IR spectroscopic analysis revealed the successful conjugation of PGE_2_, as revealed by the presence of a peak at 1,609 cm^−1^, confirming the presence of the C=N bond in the structure, whereas the band at 1,700 cm^−1^ corresponded to the C=O bonds in the carboxyl groups of PGE_2_. Furthermore, the carbonyl (∼1,726 cm^−1^) vibrations of PGE_2_ disappeared in the spectra of PEI-HBA-PGE_2_, demonstrating that hydrazone bonds were formed and that no free PGE_2_ existed in addition to PEI-HBA-PGE_2_ (Figure 1B). Then, the PEI-HBA-PGE_2_ conjugates were further cross-linked to collagen by dehydration (Figure S1C). The extent of cross-linking was further confirmed by tris-borate-EDTA-PAGE, an electrophoresis technique optimized to detect free PEI-HBA-PGE_2_. Prior to ultrafiltration, only traces of free PEI-HBA-PGE_2_ were detected, indicating a high degree of cross-linking (Figure S2B). The PGE_2_ loading capacity of the COL-PGE_2_ matrix was 1.78 micrograms of PGE_2_ per milligram of collagen, as quantified by ELISA (Figure S2C).

Rheological methods were used to analyze the gelation properties of the matrix of PGE_2_-conjugated collagen (COL-PGE_2_ matrix). The rheological profile showing the temperature changes of the COL-PGE_2_ matrix was evaluated and compared with that of the collagen matrix without immobilization of PGE_2_ (Figure S2D). With an increase in temperature from 4°C to 40°C, the storage modulus (G′) of the COL-PGE_2_ matrix showed obvious enhancement, indicating the phase transition from a solution to a gel (Figure 1C). The gelation temperature of the COL-PGE_2_ matrix was between 26°C and 27°C, which was similar to that of the collagen matrix and free PGE_2_ in the collagen matrix, indicating that the COL-PGE_2_ matrix could transform from a liquid to a gel at body temperature *in situ*. The morphological structure of the lyophilized COL-PGE_2_ matrix was observed by scanning electron microscopy (SEM). The matrix showed homogeneous and interconnected protein filaments, the average width of which was approximately 5 μm (Figure 1D), which was similar to that of collagen or free PGE_2_ in the collagen matrix (Figure S2E).

To test whether the COL-PGE_2_ matrix results in the release of PGE_2_, we investigated the release kinetics of the COL-PGE_2_ matrix and free PGE_2_ in collagen by ELISA. Free PGE_2_ in collagen was rapidly released within the first two to three days, whereas the COL-PGE_2_ matrix demonstrated prolonged release that lasted more than 16 days *in vitro* (Figure 1E). To investigate the PGE_2_ release curve of the COL-PGE_2_ matrix *in vitro*, a CCK-8 assay was used to determine the optimal concentration (2 μM) of the COL-PGE_2_ matrix that can noticeably promote cell proliferation (Figure 1F). The *in vivo* release of PGE_2_ from the COL-PGE_2_ matrix showed that the level of PGE_2_ increased incrementally after injection, whereas unbound PGE_2_ was released rapidly in the first 3 days (Figure 1G).

### Subcapsular delivery of the COL-PGE_2_ matrix exerts superior therapeutic effects

**Figure 2:**
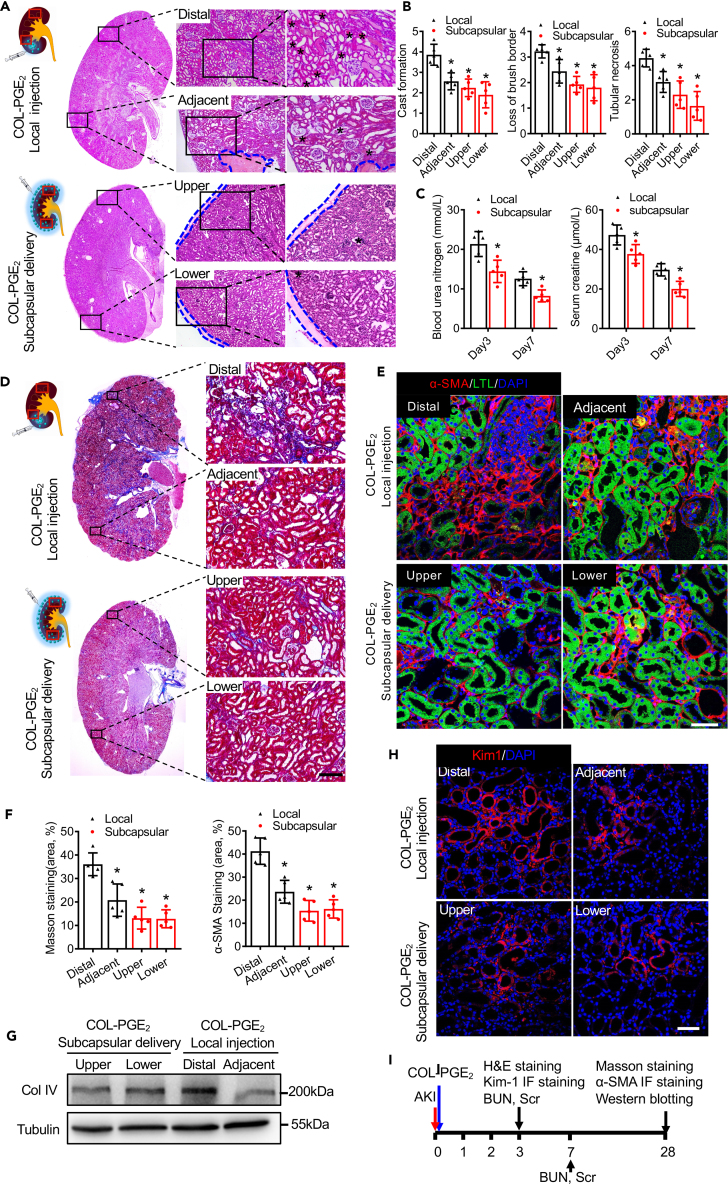
Subcapsular delivery of the COL-PGE_2_ matrix is superior to local injection in kidney recovery (A) Histological analysis of kidney injury by H&E staining on day 3 post-AKI. Top: COL-PGE_2_ matrix local injection; bottom: COL-PGE_2_ matrix subcapsular delivery. Massive necrosis is observed in proximal tubules with hyaline casts (asterisks), and subcapsular delivery of the COL-PGE_2_ matrix is superior to local injection for kidney recovery. The blue dotted lines indicate the edges of the COL-PGE_2_ matrix. Rectangles in schema represent Distal, Adjacent (upper panel) and Upper, Lower (lower panel), respectively. Scale bars, 100 μm. (B) Quantification of typical pathological changes, such as cast formation, tubular necrosis, and loss of the brush border. (C) Serum blood levels of urea nitrogen (left) and creatinine (right) were measured at different time points after AKI. (D) Representative images of kidney sections stained with Masson trichrome stain on day 28 after AKI. Rectangles in the schema represent Distal, Adjacent (upper panel) and Upper, Lower (lower panel), respectively. Scale bars, 100 μm. (E) Representative images of α-SMA (red) immunofluorescence staining on day 28 after AKI. The proximal tubules were co-stained with Lotus Tetragonolobus Lectin (LTL; green). Scale bars, 50 μm. (F) Quantitative analysis of Masson trichrome staining (left) and the α-SMA^+^ staining area (right) were performed to evaluate renal fibrosis. (G) Immunoblot analysis of type IV collagen (Col IV) protein in the kidney on day 28 after AKI. (H) Representative images of Kim-1 (red) immunofluorescence staining on day 3 after AKI. Scale bars, 50 μm. (I) Schematics of the time lines for each experiment. One-way repeated measures ANOVA with Tukey post hoc tests (B, C, and G) was used for statistical analysis. Data are expressed as mean ± SD; n = 5, ∗p < 0.05 versus distal positions of the local injection of the COL-PGE_2_ matrix local injection.

Local delivery results in a high level of drug deposition and poor local retention (Segura-Ibarra et al., 2017). To determine the therapeutic efficacy of renal subcapsular delivery, we investigated the therapeutic efficacy of the COL-PGE_2_ matrix in a mouse AKI model. First, the results of histological examination showed that necrotic tubule and hyaline cast were significantly reduced by subcapsular delivery compared with local injection of the COL-PGE_2_ matrix. Specifically, subcapsular delivery of the COL-PGE_2_ matrix significantly reduced the damage caused by AKI throughout the entire kidney, whereas local injection of the COL-PGE_2_ matrix only repaired renal tissue near the injection site (adjacent), and locations further away from the injection sites (distal) still showed severe damage (Figures 2A and 2B). Additionally, to assess functional recovery after AKI, blood urea nitrogen (BUN) and serum creatinine (Scr) were detected on day 3 and 7. We found that subcapsular delivery of the COL-PGE_2_ matrix accelerated kidney functional recovery compared with local injection, as shown by the lower values of BUN and Scr (Figure 2C).

During the later phase of kidney injury, renal fibrosis generally leads to progressive dysfunction, which can ultimately result in end-stage renal disease (Feng et al., 2016; Zhang et al., 2020). Therefore, we performed Masson trichrome staining and anti-α-Smooth muscle actin (α-SMA) immunostaining to assess renal fibrosis on day 28 after AKI. Quantification of the fibrotic area showed that subcapsular delivery of the COL-PGE_2_ matrix significantly alleviated renal fibrosis in the entire kidney compared with local injection (Figures 2D and 2F). Anti-α-SMA immunostaining also confirmed that subcapsular delivery of the COL-PGE_2_ matrix could alleviate renal fibrosis (Figures 2E and 2F). In addition, the histological results were further confirmed by western blotting analysis of collagen type IV (a marker of fibrosis) (Figure 2G). Furthermore, the evaluation of a kidney injury marker (Kim-1) by immunostaining revealed consistent results (Figure 2H). Schematics of the time lines for each experiment are shown in Figure 2I. In conclusion, compared with local injection, subcapsular delivery of the COL-PGE_2_ matrix exhibited superior outcomes in improving renal function by attenuating renal injury and suppressing renal fibrosis. Meanwhile, these results also proved the feasibility of subcapsular delivery of the COL-PGE_2_ matrix for kidney therapy.

### PGE_2_ promotes kidney repair through Yap-mediated activation of Sox9

Based on the great repair effect of PGE_2_ on the injured kidney, we investigated the therapeutic mechanism of the COL-PGE_2_ matrix in AKI. Our results confirmed that PGE_2_ could promote cell proliferation by Ki-67 staining (Figures S3A and S3B). Sox9 is a key factor in kidney regeneration (Kang et al., 2016; Zhang and Li, 2020). Interestingly, its upstream regulator, Yap, also plays an important role during animal development and regeneration (Chen et al., 2018; Moya and Halder, 2019). To explore the potential role of their interaction in kidney repair, in the present study, the western blotting analysis results showed that the COL-PGE_2_ matrix significantly increased the expression of Yap and downstream targets of amphiregulin (Areg, an EGFR ligand, promotes epithelial regeneration) (Chen et al., 2018; Zaiss et al., 2015), Survivin (an antiapoptotic molecule in renal cells) (Chen et al., 2013; Kindt et al., 2008), and Sox9. Large tumor suppressor kinase 1 (Lats1) is the major negative regulator of Yap, which sequesters Yap in the cytoplasm by phosphorylating it at the Ser127 residue. However, PGE_2_ did not affect the ratio of phosphorylated Yap. In addition, PGE_2_ did not affect Lats1 phosphorylation of Lats1 (Figure S3C). Furthermore, we investigated the relationship between Yap and Sox9 activity, and a positive correlation between Yap and Sox9 activity was observed in the group treated with the COL-PGE_2_ matrix (Figures S3D and S3E).

**Figure 3:**
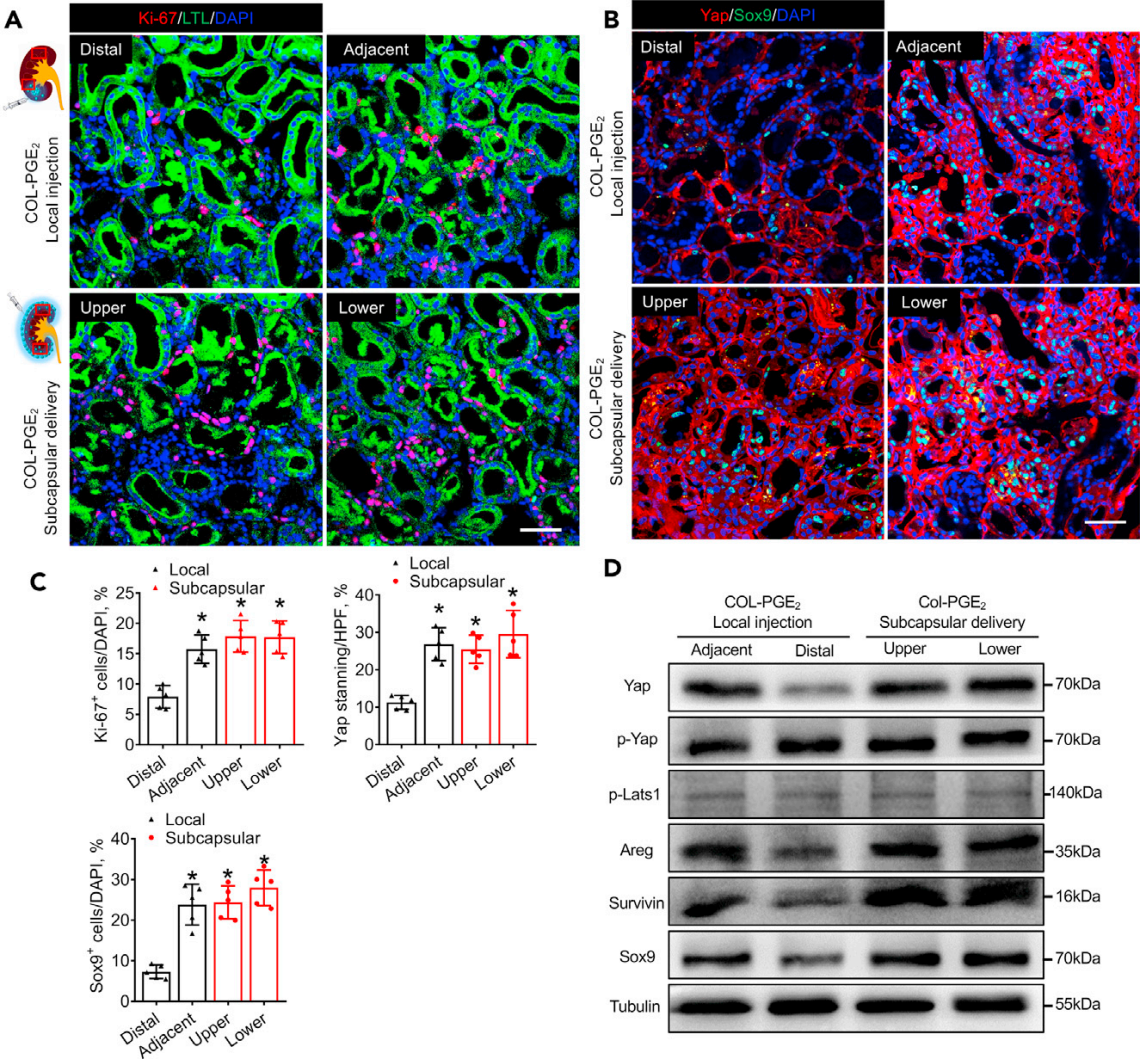
Subcapsular delivery of the COL-PGE_2_ matrix stimulates Yap-mediated expression of Sox9 in the whole kidney (A) Representative images show Ki-67 immunostaining (red) detected in renal tubular epithelial cells (FITC-labeled LTL, green) 3 days after injury. Rectangles in the schema represent Distal, Adjacent (upper panel) and Upper, Lower (lower panel), respectively. Scale bars, 50 μm. (B) Immunofluorescence staining of Yap and Sox9 in renal tubular epithelial cells on day 3 after AKI with subcapsular/local delivery of the COL-PGE_2_ matrix. Scale bars, 50 μm. (C) Quantitative analysis of the immunostaining of Ki-67, Yap, and Sox9 immunostaining. (D) Immunoblotting analysis of the Yap, p-Yap, p-Lats1, Areg, Survivin, and Sox9 protein in kidneys treated with subcapsular/local injection of the COL-PGE_2_ matrix on day 3 after AKI. One-way repeated measures ANOVA with Tukey post hoc tests (C) was used for statistical analysis. Data are expressed as mean ± SD; n = 5, ∗p < 0.05 versus distal positions of the local injection of the COL-PGE_2_ matrix local injection. HPF, high-power field.

In addition, compared with subcapsular delivery of the COL-PGE_2_ matrix, local injection resulted in an increase in Ki-67^+^ cells near the injection site, but this beneficial effect was not observed throughout the kidney (Figures 3A and 3C). Furthermore, subcapsular delivery increased the expression of Yap and its target genes Areg, Survivin, and Sox9 throughout the entire kidney, whereas local injection of the COL-PGE_2_ matrix only increased its expression in the location near the injected site (Figures 3B–3D). These results indicated that the therapeutic contributions of the COL-PGE_2_ matrix to renal repair include stimulation of endogenous cell proliferation and anti-apoptosis through Yap-mediated Sox9 activation.

### Enhanced therapeutic effects of the PGE_2_-releasing matrix

**Figure 4:**
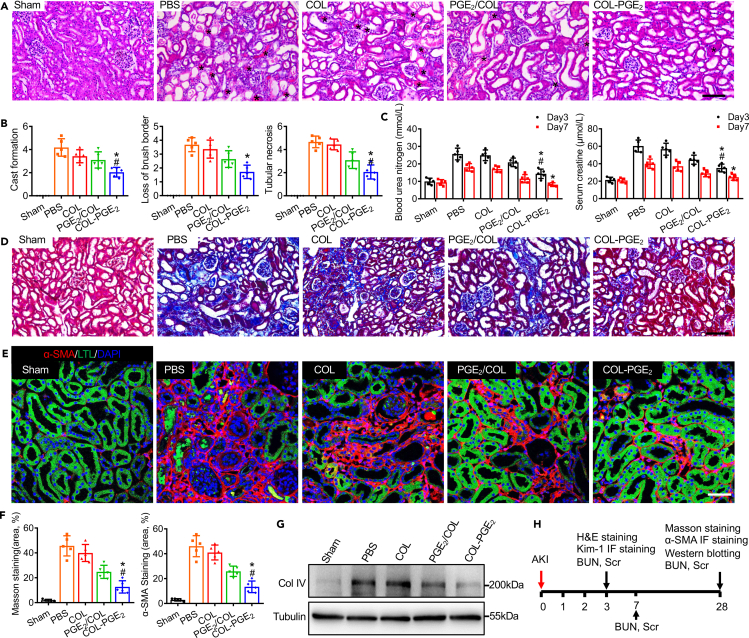
The COL-PGE_2_ matrix attenuates renal fibrosis and accelerates renal recovery (A) Representative images of the histological analysis of kidney injury by H&E staining on day 3 after treatment with the PBS, COL, PGE_2_/COL, and COL-PGE_2_ matrices after AKI. Massive necrosis was observed in the proximal tubules with hyaline casts (asterisks), and subcapsular delivery of the COL-PGE_2_ matrix revealed to have almost complete prevention of histopathological alterations after AKI. Scale bars, 100 μm. (B) Quantitative evaluations of cast formation, tubular necrosis, and injured tubules. (C) Serum blood levels of urea nitrogen (left) and creatinine (right) were measured at different time points after AKI. (D) Representative images of Masson trichrome staining of renal tissues harvested on day 28 after AKI. Scale bars, 100 μm. (E) Representative images of anti-α-SMA immunostaining (red) of renal tissues harvested on day 28 after AKI. Proximal tubules were co-stained with FITC-labeled LTL (green). Scale bars, 50 μm. (F) Quantitative analysis of Masson trichrome staining (left) and the α-SMA^+^ staining area (right) was performed to evaluate renal fibrosis. (G) Immunoblotting analysis of type IV collagen (Col Ⅳ) in the kidney on day 28 after AKI. (H) Schematics of the time lines for each experiment. One-way repeated measures ANOVA with Tukey post hoc tests (B, C, and F) was used for statistical analysis. Data are expressed as mean ± SD; n = 5, ∗p < 0.05 versus COL; ^#^p < 0.05 versus PGE_2_/COL.

We confirmed that renal subcapsular delivery of the COL-PGE_2_ matrix exhibited a greater advantage in improving renal function compared with local injection. To test whether sustained release improved the therapeutic efficacy of PGE_2_, a murine model of AKI was applied. We delivered PBS, collagen type I (COL), free PGE_2_ with collagen type I (PGE_2_/COL), and COL-PGE_2_ matrix (PGE_2_-conjugated collagen matrix) through the renal capsule. Histological changes in the early stage (day 3 post-AKI) were evaluated by hematoxylin and eosin (H&E) staining. Massive tubular cell necrosis and hyaline cast formation were observed in the proximal tubules of the kidneys in the PBS group, whereas these pathological phenomena were significantly less common in the COL-PGE_2_ matrix group (Figures 4A and 4B). Next, we evaluated the expression of Kim-1 by immunostaining. Compared with the other three groups (PBS, COL, and PGE_2_/COL), the group treated with the COL-PGE_2_ matrix showed fewer Kim-1^+^ renal tubules on day 3 post-AKI (Figures S4A and S4B). In addition, BUN and Scr were measured on day 3 and 7 post-AKI to assess renal function. The increasing concentrations of BUN and Scr reflected the deterioration of renal function. Administration of the COL-PGE_2_ matrix markedly improved renal function compared with the other three groups, manifested as a reduction in BUN and Scr levels (Figure 4C). Masson trichrome staining demonstrated a significant reduction in fibrotic area in the COL-PGE_2_ matrix group compared with the other groups (Figures 4D and 4F). α-SMA staining also demonstrated findings similar to Masson trichrome staining (Figures 4E and 4F). Furthermore, histological results were supported by a type IV collagen western blot analysis (Figure 4G). Furthermore, we evaluated the degradation and safety of the COL-PGE_2_ matrix in the kidney, and the COL-PGE_2_ matrix was almost completely degraded after 28 days (Figures S4C and S4D). H&E staining was also performed; there were no pathological changes in the heart, liver, spleen, and lung in each group after treatment with the COL, PGE_2_/COL, and COL-PGE_2_ matrix on day 28 after AKI (Figure S4E). Schematics of the time lines for each experiment are revealed in Figure 4H. In conclusion, the COL-PGE_2_ matrix was superior in promoting kidney regeneration by gradually releasing PGE_2_.

### Lineage tracing of Sox9^+^ cells by intravital microscopy after COL-PGE_2_ matrix therapy

**Figure 5:**
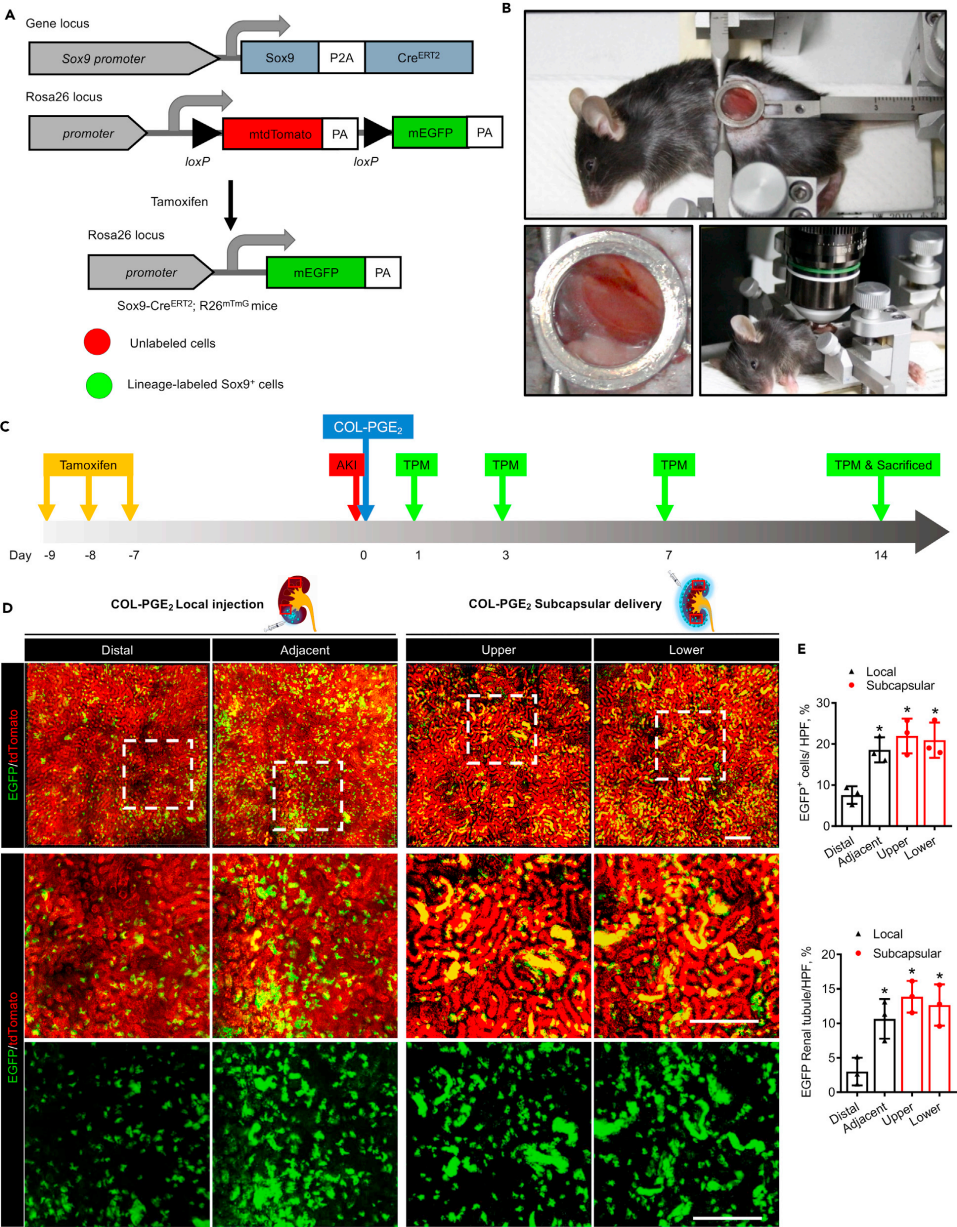
Sox9+ cell expansion under COL-PGE_2_ matrix therapy by intravital microscopy (A) Scheme of the generation of tamoxifen-inducible Sox9-Cre^ERT2^; R26^mTmG^ mice for genetic lineage tracing. Polyadenylation sequences (PA). (B) Sox9-Cre^ERT2^; R26^mTmG^ mouse with the abdominal imaging window (AIW) on the left kidney (top). Close-up view of the kidney window (left). Mouse with AIW installed for two-photon intravital microscopy analysis (right). (C) Schematic illustration of two-photon live cell imaging of Sox9^+^ cells after AKI and COL-PGE_2_ matrix administration. Sox9-Cre^ERT2^; R26^mTmG^ mice were injected intraperitoneally with tamoxifen once a day for three continuous days to label Sox9 + cells. Seven days after the final tamoxifen injection, renal ischemia-reperfusion injury was induced in these mice with simultaneous implantation of AIW. Two-photon intravital microscopy imaging was performed on days 1, 3, 7, and 14 after AKI. (D) Representative images of two-photon intravital tracing showed that Sox9^+^-cell-derived cells expanded abundantly after subcapsular delivery of the COL-PGE_2_ matrix compared with local injection on day 7 after AKI. Rectangles in the schema represent Distal, Adjacent (upper panel) and Upper, Lower (lower panel), respectively. Scale bar, 200 μm. (E) Quantification of EGFP-labeled renal tubules and Sox9^+^/EGFP co-labeled cells in the kidneys of mice on day 7 with subcapsular/local delivery of the COL-PGE_2_ matrix post-AKI. One-way repeated measures ANOVA with Tukey post hoc tests were used for statistical analysis. Data are expressed as mean ± SD; n = 3, ∗p < 0.05 versus distal positions of COL-PGE_2_ matrix local injection. See also Videos S5, S6, S7, and S8.

High-resolution intravital two-photo microscopy (TPM) offers a platform for investigating the spatiotemporal kinetics of endogenous stem cells in tissue regeneration at the single-cell level (Dondossola et al., 2018; Hato et al., 2017; Honkura et al., 2018; Zhang and Li, 2020). Recent evidence indicates that Sox9^+^ cells show progenitor-like properties and that proliferation and differentiation of Sox9^+^ endogenous renal progenitor cells are mainly responsible for renal tubular regeneration after AKI (Kang et al., 2016; Kumar et al., 2015). In this study, a mouse model of the Sox9 lineage tracing (Balani et al., 2017; Roche et al., 2015; Zhang and Li, 2020; Zhang et al., 2016), Sox9-Cre^ERT2^; R26^mTmG^, was used to monitor Sox9^+^ cells and their descendants with an abdominal imaging window (AIW) (Figures 5A–5C). Furthermore, we performed intravital lineage tracing of Sox9^+^ cells in AKI mice (Figure 5B). The results revealed that EGFP was only expressed in Sox9^+^ cells after tamoxifen induction and that there was no spontaneous AKI-induced Cre activation in Sox9-Cre^ERT2^; R26^mTmG^ mice (Figure S5).

Our results revealed that Sox9^+^ cells can be tracked with intravital TPM and that the COL-PGE_2_ matrix significantly increased the number of EGFP^+^ cells, which can form renal tubular structures on day 7 after AKI (Figure S6). In the sham operation group, all renal cells were substantially positive for tdTomato, indicating that renal cells were largely Sox9^−^ in the normal adult kidney. Compared with local injection, subcapsular delivery of the COL-PGE_2_ matrix increased the number of EGFP^+^ cells in the entire kidney and led to the obvious formation of renal tubular structures (Figure 5D and Videos S5, S6, S7, and S8). Furthermore, subcapsular delivery of the COL-PGE_2_ matrix promoted the expansion of EGFP^+^ cells throughout the entire kidney, whereas local injection increased the expansion only near the injected site (Figures 5E and S7; Videos S1, S2, S3, S4, S5, S6, S7, and S8).

Video S1. Sham surgery on day 7. 3D reconstruction of the renal structure in the Sox9-Cre^ERT2^; R26^mTmG^ mice treated by sham surgery on day 7. Scale bar, 300 μm, related to Figure S6

Video S2. Treated with PBS via subcapsular delivery on day 7 after AKI. 3D reconstruction of the renal structure in the Sox9-Cre^ERT2^; R26^mTmG^ mice treated with PBS via subcapsular delivery on day 7 after AKI. Scale bar, 300 μm, related to Figure S6

Video S3. Treated with collagen matrix via subcapsular delivery on day 7 after AKI. 3D reconstruction of the renal structure in the Sox9-Cre^ERT2^; R26^mTmG^ mice treated with collagen matrix via subcapsular delivery on day 7 after AKI. Scale bar, 300 μm, related to Figure S6

Video S4. Treated with PGE_2_/COL matrix via subcapsular delivery on day 7 after AKI. 3D reconstruction of the renal structure in the Sox9-Cre^ERT2^; R26^mTmG^ mice treated with PGE2/COL matrix via subcapsular delivery on day 7 after AKI. Scale bar, 300 μm, related to Figure S6

Video S5. Treated with COL-PGE_2_ matrix via subcapsular delivery on day 7 after AKI (upper structure). 3D reconstruction of the upper renal structure in the Sox9-Cre^ERT2^; R26^mTmG^ mice treated with the COL-PGE_2_ matrix via subcapsular delivery on day 7 after AKI. Scale bar, 300 μm, related to Figure 5

Video S6. Treated with COL-PGE_2_ matrix via subcapsular delivery on day 7 after AKI (lower structure). 3D reconstruction of the lower renal structure in the Sox9-Cre^ERT2^; R26^mTmG^ mice treated with the COL-PGE_2_ matrix via subcapsular delivery on day 7 after AKI. Scale bar, 300 μm, related to Figure 5

Video S7. Treated with COL-PGE_2_ matrix via local injection on day 7 after AKI (distal injection site). 3D reconstruction of the renal structure at the distal injection site of Sox9-Cre^ERT2^; R26^mTmG^ mice treated with COL-PGE_2_ matrix via local injection on day 7 after AKI. Scale bar, 300 μm, related to Figure 5

Video S8. Treated with COL-PGE_2_ matrix via local injection on day 7 after AKI (adjacent injection site). 3D reconstruction of the renal structure of the adjacent injection site of Sox9-Cre^ERT2^; R26^mTmG^ mice treated with COL-PGE_2_ matrix via local injection on day 7 after AKI. Scale bar, 300 μm, related to Figure 5

**Figure 6:**
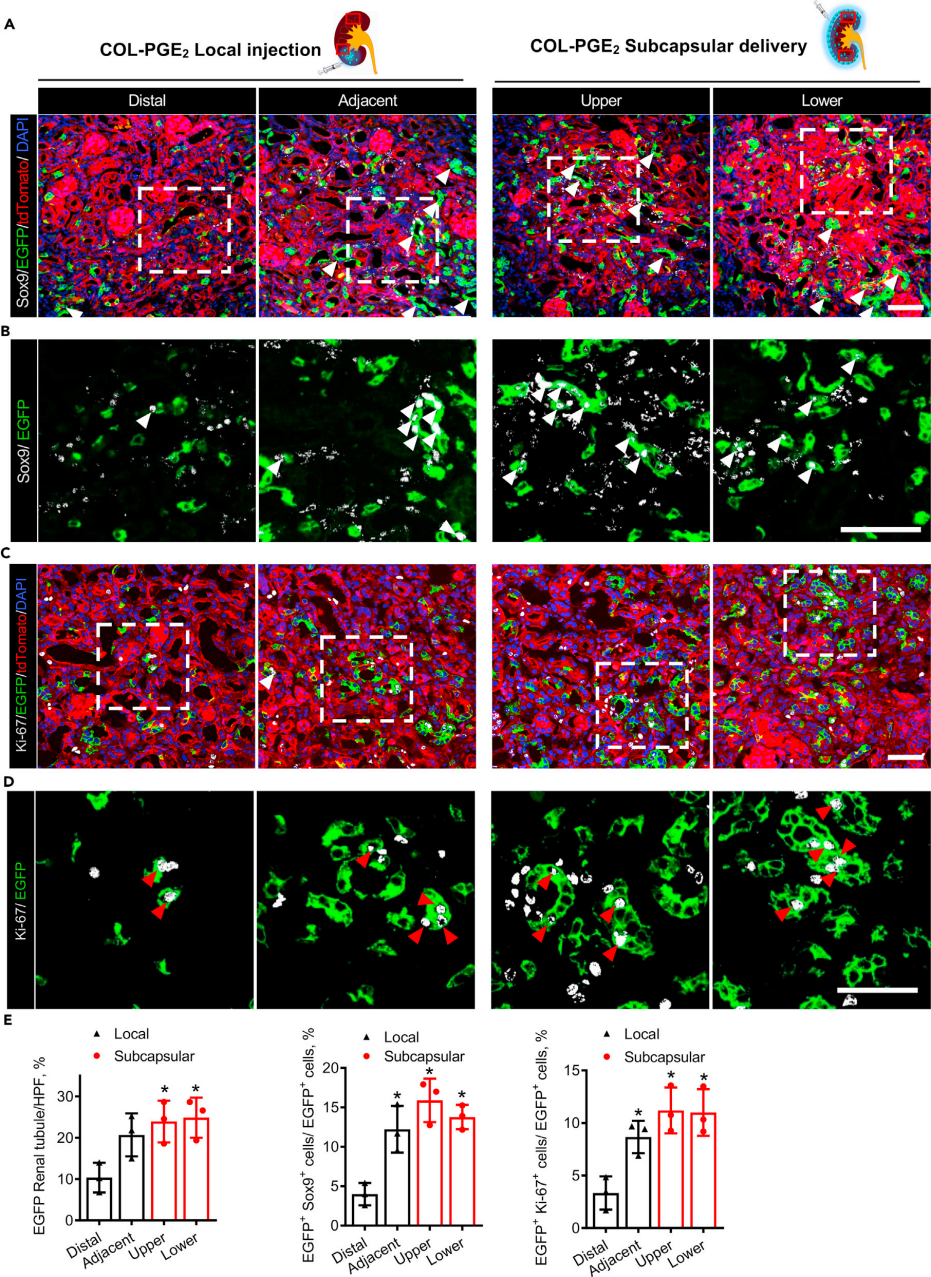
Subcapsular delivery of the COL-PGE_2_ matrix results in greater activation of Sox9 expression and Sox9^+^ cell proliferation (A and B) (A) Confocal images and (B) local zoomed-in images for colocalization analysis of anti-Sox9 immunostaining (gray) and Sox9-Cre^ERT2^-activated EGFP fluorescence in kidneys with local injection (left) and subcapsular delivery (right) of the COL-PGE_2_ matrix on day 14 post-AKI. White arrowheads highlight the renal tubules formed by Sox9-EGFP-labeled cells, and white arrowheads highlight Sox9^+^/EGFP co-labeled cells. Rectangles in the schema represent Distal, Adjacent (upper panel) and Upper, Lower (lower panel), respectively. Scale bar, 50 μm. (C and D) (C) Confocal images and (D) local magnification images for colocalization analysis of anti-Ki-67 immunostaining (gray) and Sox9-Cre^ERT2^-activated EGFP fluorescence in kidneys with local injection (left) and subcapsular delivery (right) of the COL-PGE_2_ matrix on day 14 post-AKI. Red arrowheads highlight Ki-67^+^/EGFP^+^ co-labeled cells. Scale bar, 50 μm. (E) Quantification of EGFP^+^ renal tubules, Sox9^+^/EGFP^+^ co-labeled cells, and Ki-67^+^/EGFP^+^ co-labeled cells in the kidneys of mice on day 14 after subcapsular or local delivery of the COL-PGE_2_ matrix post-AKI. One-way repeated measures ANOVA with Tukey post hoc tests (E) was used for statistical analysis. Data are expressed as mean ± SD; n = 3, ∗p < 0.05 versus distal positions of the local injection of the COL-PGE_2_ matrix local injection.

To investigate Sox9 activity under the treatment of the COL-PGE_2_ matrix, we further performed an anti-Sox9 immunostaining analysis of kidney samples collected from Sox9-Cre^ERT2^; R26^mTmG^ mice on day 14 after the last round of TPM imaging. In normal adult kidneys, EGFP^+^ cells were rare, and most of the cells were Sox9^−^ (Figure S8); this suggested that only a small fraction of renal cells was derived from Sox9^+^ cells under physiological conditions. Post-AKI, renal injury promoted Sox9 expression and stimulated the expansion of EGFP^+^ cells, but most descendants of EGFP^+^ cells no longer expressed Sox9. It was intriguing that the COL-PGE_2_ matrix promoted the expansion of EGFP^+^ cells and maintained their expression of Sox9 for a period of time (Figures 6A, 6B, and 6E). Additionally, subcapsular delivery of the COL-PGE_2_ matrix stimulated Sox9 expression and cell proliferation (Ki-67) compared with local injection (Figures 6C–6E). Taken together, these findings indicated that the COL-PGE_2_ matrix promoted the expansion of EGFP^+^ cells while maintaining their progenitor cell characteristics and stimulated more cells to express Sox9 after enhancing their proliferation capacity. The COL-PGE_2_ matrix also boosted the formation of functional renal tubules by promoting the proliferation of Sox9^+^ cell descendants.

### Cytoprotective effects of the COL-PGE_2_ matrix

**Figure 7:**
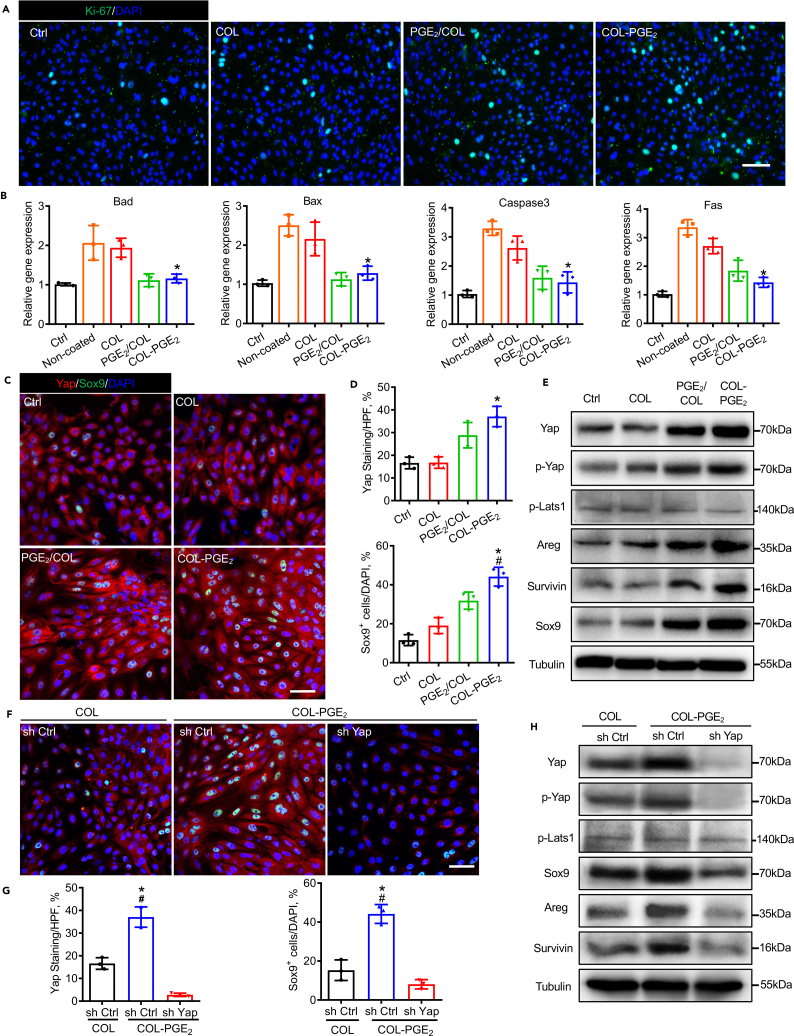
The COL-PGE_2_ matrix favors renal tubular epithelial cell proliferation and cytoprotection *in vitro* (A) Immunofluorescence staining of Ki-67 (green) in primary renal tubular epithelial cells (pRTECs) cultured on plates coated with 2.0 μM COL-PGE_2_, PGE_2_/COL, or COL matrix or uncoated plates. Scale bars, 100 μm. (B) Expression levels of apoptosis-related genes were evaluated by qPCR. (C) Immunofluorescence staining of Yap (red) and Sox9 (green) in pRTEC cultured in noncoated, COL, PGE_2_/COL, or COL-PGE_2_ at 48 h. Scale bars, 50 μm. (D) Quantification of the Yap^+^ area and Sox9^+^ cells in (C). (E) Immunoblot analysis of the Yap, p-Yap, p-Lats1, Areg, Survivin, and Sox9 protein in pRTEC cultured on noncoated, COL, PGE_2_/COL, or COL-PGE_2_ matrix at 48 h. β-tubulin was used as loading control. (F) Immunofluorescence staining of Yap (red) and Sox9 (green) in pRTEC cultured in plates coated with COL or COL-PGE_2_ and pRTECs with YAP knockdown cultured on plates at 48 h. Scale bars, 50 μm. (G) Quantification of the Yap^+^ area and Sox9^+^ cells in (F). (H) Western blot analysis of the Yap, p-Yap, p-Lats1, Areg, Survivin, and Sox9 protein in pRTEC cultured on COL- or COL-PGE_2_-coated plates and pRTEC with Yap knockdown cultured on plates at 48 h. β-tubulin was used as loading control. One-way repeated measures ANOVA with Tukey post hoc tests (B and D) was used for statistical analysis. Data are expressed as mean ± SD; n = 3. ∗p < 0.05 versus COL; ^#^p < 0.05 versus PGE_2_/COL. One-way repeated measures ANOVA with Tukey post hoc tests (G) was used for statistical analysis. Data are expressed as mean ± SD; n = 3. ∗p < 0.05 versus the non-targeting shRNA (sh Ctrl) cultured in COL; ^#^p < 0.05 versus the shRNA targeting YAP (sh-Yap) cultured in COL-PGE_2_.

To verify the mechanism of the PGE _2_ matrix in kidney repair, we isolated primary renal tubular epithelial cells (pRTECs) from C57BL/6 mice, which were confirmed by cell morphology and immunofluorescence staining of E-cadherin and Aquaporin-1 (Figures S9A and S9B). The morphology and colony-forming capacity of pRTEC and trypan blue stain revealed the superior biocompatibility of the COL-PGE_2_ matrix (Figures S9C and S9D). Immunofluorescence results showed that the expression of the proliferation-related gene Ki-67 was drastically upregulated in pRTEC cultured on COL-PGE_2_-matrix-coated plates compared with that in pRTECs cultured on PGE_2_/COL-matrix-coated, COL-matrix-coated, or noncoated plates (Figure 7A). To examine the cell protective effects of the COL-PGE_2_ matrix, pRTECs were cultured in plates coated with COL, PGE_2_/COL, and COL-PGE_2_ matrix and treated with hydrogen peroxide (H_2_O_2_). Staining of apoptotic cells with Hoechst 33,342 demonstrated that the PGE_2_/COL and COL-PGE_2_ matrix significantly reduced the apoptosis of pRTECs induced by H_2_O_2_ (Figure S10). To determine the pathway involved in this protective action, we measured the expression of genes in pRTEC after treatment with H_2_O_2_ (150 μM) for 6 h. Treatment with H_2_O_2_ increased the expression of the apoptosis-associated genes Bad, Bax, Caspase-3, and Fas in pRTEC. Coating plates with the PGE_2_/COL and COL-PGE_2_ matrix significantly reduced the expression of these genes (Figure 7B). Additionally, the COL-PGE_2_ matrix stimulated pRTEC proliferation and survival of pRTECs through the Yap-mediated Areg and Survivin pathway and promoted the activation of Sox9, a marker of renal progenitor cell (Figures 7C–7E). Unlike the activation of Yap by the COL-PGE_2_ matrix, PGE_2_ failed to activate Sox9 in pRTEC with Yap depletion. Furthermore, depletion of Yap by shRNA resulted in a reduction of Areg and Survivin, even though pRTECs were treated with the COL-PGE_2_ matrix (Figures 7F–7H). In conclusion, the COL-PGE_2_ matrix favors the proliferation of renal tubular epithelial cells and exerts cytoprotective effects *in vitro*.

## Discussion

This study demonstrates that renal subcapsular delivery of the COL-PGE_2_ matrix effectively improves functional recovery after AKI. The COL-PGE_2_ matrix results in an adequate long-term release of PGE_2_ in the kidney with extensive intraparenchymal penetration and maintenance of PGE_2_ release. This approach overcomes the limitations of conventional local free drug delivery, which cannot provide sustained drug release for long periods and results in insufficient distribution within the whole kidney. Sox9 cell lineage tracing with intravital microscopy revealed that PGE_2_ could significantly ameliorate kidney function by activating the renal endogenous progenitor Sox9^+^ cells through the Yap signaling pathway. We highlight the potential of COL-PGE_2_ matrix administration in the perirenal space as a viable strategy for local and sustained delivery in kidney disease therapy.

PGE_2_ has long been recognized to play a role in immune regulation and tissue regeneration (Martinez-Colon and Moore, 2018; Nasrallah et al., 2016; Palla et al., 2021). However, its short half-life time *in vivo* limits the application of PGE_2_. Several strategies, such as inhibition of the PGE_2_ catabolizing enzyme, covalent coupling of PGE_2_ with prodrugs, or incorporation of PGE_2_ into biomaterials to prolong release, have been applied in regenerating therapy (Young and Grynpas, 2018; Zhang et al., 2015, 2018). To our knowledge, this is the first study of immobilization of PGE_2_ within a collagen matrix for endogenous progenitor activation. Slow release of bioactive small molecules through covalent coupling to the matrix is an attractive strategy to enhance tissue regeneration after injury or damage and to ensure safety and effectiveness. Our findings highlight the potential use of the COL-PGE_2_ matrix as a novel therapeutic strategy to promote renal tissue regeneration through subcapsular transplantation.

Previous investigations have shown that administering drugs in the peri-organ space of organs such as the heart and kidney provides a strategy to utilize a site-specific prolonged drug release reservoir to target organs at high local concentrations (Garcia et al., 2017; Segura-Ibarra et al., 2017; Soranno et al., 2016). The unique space of the perirenal sac makes it an ideal platform for drug elution on the surface of the kidney, avoiding tissue damage by local injection and a lack of drug distribution to positions farther away from injection sites. Our results reveal the drawback of local PGE_2_ delivery, which leads to limited improvement in renal function. Covalent cross-linking of growth factors, peptides, or small molecules with biomaterials, such as collagen matrix, polymers, dendrimers, and chitosan scaffolds, produces an injectable and stabilized matrix for the delivery of pro-survival factor in tissue injury therapy (Feng et al., 2016; Lee et al., 2018; Zhang and Li, 2020; Zhao et al., 2019). In this study, an injectable PGE_2_ collagen matrix was generated by cross-linking collagen with PGE_2_ through polyethyleneimine (PEI). Furthermore, the hydrazone bond formed between PGE_2_ and PEI is an unstable covalent bond, and the collagen matrix will serve as a reservoir of PGE_2_ for prolonged release. In summary, we have demonstrated the safety and feasibility of our renal capsular delivery method according to the standards of the Food and Drug Administration and have shown that it is compatible with PGE_2_ and collagen, providing an example of its translational application to AKI therapy.

Stem cell therapy holds great promise for kidney regeneration after AKI (Fazekas and Griffin, 2020; Feng et al., 2016; James et al., 2020; Zaw Thin et al., 2020). However, acute cell death, immune responses, oncogenic properties, and ethical concerns limit the use of stem cells in clinical practice (Choumerianou et al., 2008), and few such therapies have succeeded in clinical practice (Lumelsky et al., 2018). The strategy based on optimizing endogenous tissue responses and activation of resident stem cells will enhance tissue healing and regeneration (Lumelsky et al., 2018; Tremblay et al., 2020). Tissue-resident stem cells in specific microenvironments are tightly regulated by extrinsic and intrinsic factors of cells for tissue homeostasis and regeneration (He et al., 2014; Liu et al., 2016; Lumelsky et al., 2018). Targeting the repair pathway by activating endogenous stem cells will offer a framework for AKI therapy (Humphreys et al., 2016; Zhang et al., 2020; Zhang and Li, 2020). Sox9^+^ cells have been identified as endogenous renal progenitor cells, and studies indicate that proliferation and differentiation of Sox9^+^ cells are primarily responsible for renal tubular regeneration and renal repair after AKI (Kang et al., 2016; Kumar et al., 2015). Yap activation has been shown to play an important role in renal repair after injury (Moya and Halder, 2019; Xu et al., 2016). Yap regulates the transcription of Sox9 through a conserved TEAD binding site in the Sox9^+^ promoter (Song et al., 2014). We found that the COL-PGE_2_ matrix could promote the proliferation of endogenous Sox9 progenitor cells by increasing the expression of the Yap protein and the number of Sox9^+^ cells. Our results highlight that PGE_2_ could activate endogenous Sox9^+^ renal stem cells and reveal a potential therapeutic approach for AKI therapy. Advances in imaging-based monitoring methods have allowed the tracking of stem cells and the evaluation of their therapeutic effects (Huang et al., 2015; Liu et al., 2020; Zhang and Li, 2020). Intravital two-photon microscopy (TPM) with an abdominal imaging window (AIW) allows monitoring of the spatiotemporal dynamics of endogenous stem cells in the processes of tissue repair at the single-cell level in living animals longitudinally for weeks using a lineage tracing animal model (Hato et al., 2017; Rakhilin et al., 2019; Zhang and Li, 2020). In this study, Sox9^+^ cell tracking provided new insights into the dynamic cellular processes involved in kidney regeneration with high resolution under treatment with the COL-PGE_2_ matrix. Intravital lineage tracing offers a novel strategy for exploring the therapeutic mechanism of resident cells in tissue regeneration of the kidney and other organs.

In summary, we developed a novel method to deliver the PGE_2_-releasing matrix to the kidney through the perirenal sac. Many types of gel can be used with this platform, provide a new treatment strategy for kidney diseases, and may be particularly useful for the delivery of small molecular drugs, peptides, cytokines, miRNAs, and stem cells. By taking advantage of the adequate space and proximity of the renal parenchyma, renal subcapsular delivery is a minimally invasive and effective delivery technique for the entire kidney that will aid in the translation of drug-releasing biomaterials into the clinic for kidney diseases. Furthermore, the activation of endogenous Sox9^+^ cells by PGE_2_ with the lineage tracking method and intravital microscopy highlights the therapeutic effects of PGE_2_ on tissue regeneration. Future studies are needed to better understand the underlying mechanisms of PGE_2_ and optimize the COL-PGE_2_ matrix system to help develop new methods for regenerative therapy. Overall, our study of the COL-PGE_2_ matrix established a proof-of-concept for a therapeutic that is feasible and effective in improving AKI by activating resident stem cells of the kidney.

### Limitations of the study

Renal subcapsular delivery presents many advantages over existing drug delivery methods that will aid in the translation of drug-releasing biomaterials into the clinic for kidney diseases. The application of subcapsular injection in clinical treatment still needs to explore more suitable methods for transplantation. In addition, PGE_2_ activates the differentiation of endogenous Sox9^+^ cells differentiation into renal tubular epithelial cells and other kidney cells and is critically involved in kidney repair. However, it is not clear about the specific differentiation direction and tendency of Sox9^+^ cells after PGE_2_ treatment. Thus, more work is needed to understand how PGE_2_-induced Yap regulates the specific differentiation of Sox9^+^ cells and which patients could benefit from Sox9 activation.

## STAR★Methods

### Key resources table

**Table:** 

REAGENT or RESOURCE	SOURCE	IDENTIFIER
**Antibodies**		

anti-Yap(H-9)	Santa Cruz Biotechnology	Cat#sc-271134; RRID: AB_10612397
anti-Phospho-YAP (Ser127)	Cell Signaling Technology	Cat#13008; RRID: AB_2650553
anti-Sox9 (D8G8H)	Abcam	Cat#ab185966; RRID: AB_2728660
anti-Ki-67(D3B5)	Cell Signaling Technology	Cat#12075; RRID: AB_2728830
anti-Kim-1	Abcam	Cat#ab47635; RRID: AB_882998
anti-α-SMA	BOSTER	Cat#BM0002; RRID: AB_2811044
anti-CD31	BD Biosciences Pharmingen	Cat#550274; RRID: AB_393571
anti-Areg	Abcam	Cat#ab180722
anti-Survivin(D-8)	Santa Cruz	Cat#sc-17779; RRID: AB_628302
anti-Phospho-LATS1 (Thr1079)	Cell Signaling Technology	Cat#8654; RRID: AB_10971635
anti-COL IV	Abcam	Cat#ab19808; RRID: AB_445160
anti-β-tubulin	Proteintech Group	Cat#10068-1-AP; RRID: AB_2303998

**Chemicals, peptides, and recombinant proteins**		

PGE_2_	Santa Cruz	Cat#sc-201225
Collagen I, Rat Tail	Corning	Cat#354236
4-hydrazinobenzoic acid	Molbase	Cat#SJ000FFC61940
Polyethylenimine	Polysciences, Inc	Cat#19850
EDC (1-ethyl-3-[3-dimethylaminopropyl]carbodiimide hydrochloride)	Thermo Scientific	Cat#22980
sulfo-NHS(N-hydroxysulfosuccinimide)	Thermo Scientific	Cat#24510
TRIzol reagent	Invitrogen	Cat#15596018
RIPA lysis buffer	Solarbio	Cat#R0010
Hieff® qPCR SYBR® Green Master Mix (No Rox)	Yeasen	Cat#11201ES08
FITC-labeled LTL	Vector Laboratories	Cat# FL-1321-2
DAPI	Beyotime Biotechnology	Cat#C1002
2-(N-morpholino) ethanesulfonic acid	Sangon Biotech	Cat#145224

**Biological samples**		

primary renal tubular epithelial cells	This Paper	N/A
Transgenic mice for C57BL/6 Rosa26^mTmG^ transgenic mice	Jackson Laboratory	Cat#007676
C57BL/6 Sox9-Cre^ERT2^ transgenic mice	Jackson Laboratory	Cat#018829
C57BL/6 mice	Laboratory Animal Center of the Academy of Military Medical Science	N/A

**Critical commercial assays**		

PGE_2_ Enzyme Immunoassay Kit	ArborAssays	Cat#K051-H1
Urea Assay Kit	Nanjing Jiancheng Bioengineering Institute	Cat#C013-1
Creatinine Assay Kit	Nanjing Jiancheng Bioengineering Institute	Cat#C011-1
Cell Counting Kit-8	Beyotime Biotechnology	Cat#C0038
Reverse transcriptase core kit	Takara	Cat#RR037A
Hoechst Staining Kit	Beyotime Biotechnology	Cat#C0003

**Oligonucleotides**		

*Bad* 5′-AAGTCCGATCCCGGAATCC-3′	This paper	N/A
*Bad* 5′-GCTCACTCGGCTCAAACTCT-3′	This paper	N/A
*Bax* 5′-TGAAGACAGGGGCCTTTTTG-3′	This paper	N/A
*Bax* 5′-AATTCGCCGGAGACACTCG-3′	This paper	N/A
*Caspase3* 5′-TGGTGATGAAGGGGTC ATTTATG-3′	This paper	N/A
*Caspase3* 5′-TTCGGCTTTCCAGTCAGA CTC-3′	This paper	N/A
*Fas* 5′-TATCAAGGAGGCCCATTTTGC-3′	This paper	N/A
*Fas* 5′-TGTTTCCACTTCTAAACCATGCT-3′	This paper	N/A
*Gapdh* 5′-AGGTCGGTGTGAACGGATTTG-3′	This paper	N/A
*Gapdh* 5′-TGTAGACCATGTAGTTGAGG TCA-3′	This paper	N/A
*Nonspecific shRNA (sh Ctrl)* 5′-CCGG-CA ACAAGATGAAGAGCACCAA-CTCGAG- TTGGTGCTCTTCATCTTGTTG-TTTTTG-3′	Sigma-Aldrich	Cat#SHC002
*shRNA-Yap (sh Yap)* 5′-CCGG-GCAGAC AGATTCCTTTGTTAA-CTCGAG-TTAAC AAAGGAATCTGTCTGC-TTTTTG-3′	Broad Institute	TRCN0000095864

S**oftware and algorithms**		

Two-photon microscope system	Olympus	FV1000
Scanning electron microscopy (SEM)	HITACHI	X-650
FT-IR spectromete	Bio-Rad	FTS-6000
Parallel-plate rheometer	New Castle	N/A
Imaris	Oxinst	https://imaris.oxinst.com/
Prism 8	GraphPad	https://www.graphpad.com/scientific-software/prism/
ImageJ	ImageJ	https://imagej.nih.gov/ij

### Resource availability

#### Lead contact

Further information, inquiries, and request should be directed to the lead contact Zongjin Li (zongjinli@nankai.edu.cn).

#### Materials availability

All requests for resources and reagents should be directed to and will be fulfilled by the lead contact Zongjin Li (zongjinli@nankai.edu.cn). All reagents will be made available on request after completion of a Materials Transfer Agreement.

### Experimental model and subject details

#### AKI model and COL-PGE_2_ matrix delivery

Adult male wild-type C57BL/6 mice (7-8 weeks old, weight 25-30 g) were purchased from the Laboratory Animal Center of the Academy of Military Medical Sciences (Beijing, China). Animals were randomly assigned to six groups according to the complete randomization method, and each group included five single mice. An AKI model induced by ischemia/perfusion was established as previously described (Liu et al., 2018). Briefly, the animals were anesthetized by intraperitoneal injection of 2.5% avertin (Sigma-Aldrich) at a dose of 240 mg/kg. Mice were clamped to the left kidney pedicle for 40 min followed by removal of the right kidney after injury. Reperfusion was visually confirmed before delivery of the matrix. After 10 min of reperfusion, 75 μl of PBS, COL, PGE_2_/COL or COL-PGE_2_ matrix was injected into the renal capsule or the renal cortex with an insulin syringe. The kidney was kept moist with warm sterile saline prior to delivery of the matrix. The needle of the matrix-containing syringe pierced the kidney capsule at the upper end of the kidney. The needle was slowly moved from the upper end to the lower end along the inner surface of the renal capsule. The middle of the needle was pressed to slowly inject the matrix; then, the syringe was slowly pulled out and the injection site was pressed with a cotton swab for 3-5 min. The kidney was returned to the abdominal cavity after the matrix solidified and the wound was sewn. The animal was placed in an incubator until it came out from anesthesia. In addition, another group of intra-local injections of COL-PGE_2_ matrix was administered as the control group. Injection of PBS and collagen matrix was performed in controls. Sham-operated animals were subjected to the same surgical procedure without renal ischemia or matrix injection. The treatment of animals and the experimental procedures of the present study adhere to the Nankai University Animal Care and Use Committee Guidelines (approval no. IRM-DWLL-2019121), which is in accordance with the Guidelines for Animal Care Guidelines approved by the National Institutes of Health (NIH).

#### Tracing of the Sox9 lineage with transgenic mice

Male C57BL/6 Sox9-Cre^ERT2^; Rosa26^mTmG^ transgenic mice were purchased from Jackson Laboratory (Jax strains: 007676 and 018829) and have been used for lineage tracing in multiple studies (Balani et al., 2017; Roche et al., 2015; Zhang and Li, 2020; Zhang et al., 2016). Mice were randomly divided into 6 groups at random and each group included 3 single mice. Rosa26^mTmG^ mice express the fluorescent protein tdTomato (red) under the β-actin promoter. Through Cre-mediated recombination, the Tomato and STOP codons are excised, and EGFP is expressed in these animals. Sox9-Cre^ERT2^ animals express a ligand-dependent chimeric Cre recombinase under the Sox9 promoter. Cell labeling occurs only at the time of tamoxifen injection, but daughter cells retain the labels, as genetic recombination cannot be reversed in the nucleus. EGFP is expressed in a Sox9^+^ cell type-specific manner. Transgenic mice were identified by genomic PCR analysis. Only 5- to 8-week-old male mice were used in the study. Mice were injected with tamoxifen dissolved in corn oil intraperitoneally (100 mg/kg daily, mice were injected 3 times a week before the experiment).

### Method details

#### Preparation of the COL-PGE_2_ matrix

As summarized in Figure 1A, PGE_2_ was first linked to the PEI-HBA cross-linker to obtain PEI-HBA-PGE_2_ conjugates, and then the PEI-HBA-PGE_2_ conjugates were further covalently cross-linked to collagen through dehydration condensation. In detail, to increase/decrease, 4.8 μmol branched polyethylenimine (PEI, Mw = 10000, 60 mg) and 0.1 mmol of 4-hydrazinobenzoic acid (HBA, 14.8 mg) were dissolved in 5 mL of dimethyl sulfoxide (DMSO), and then 0.28 mmol 1-(3-dimethylaminopropyl)-3-ethylcarbodiimide hydrochloride (EDC, 54 mg) and 0.28 mmol N-hydroxy-succinimide (sulfo-NHS, 32 mg) were added. The mixture was stirred for 24 h at room temperature in an atmosphere of nitrogen and then the HBA-PEI conjugates were dialyzed against H_2_O to remove unreacted HBA (molecular weight cutoff [MWCO] =3,500) and lyophilized to obtain a fine powder for the next step (Figure S1A). For the cross-linking of PGE_2_, the PGE_2_ (10 mg, CAS 363-24-6; Santa Cruz Biotechnology) and PEI-HBA conjugates (30 mg) were dissolved separately in 10 mL of ethanol and mixed together, and the mixture was added to 2 mL of glacial acetic acid, stirred for 2 h, The reaction mixture was heated to 80°C, refluxed by adding molecular sieves under nitrogen for 20 h, and allowed to cool at 0°C. The product was dialyzed against H_2_O (MWCO =3,500) and lyophilized to obtain a fine powder (HBA-PEI-PGE_2_) (Figure S1B). The last step was to link the HBA-PEI-PGE_2_ conjugates to collagen (Figure S1C). To activate collagen, 5 mg of EDC and 5 mg of sulfo-NHS were added to 6 mL of collagen solution, which was dialysed against 2-(N-morpholino) ethanesulfonic acid (MES buffer, 50 mM) and reacted for 20 min at room temperature, and then 1.4 μL of 2-mercaptoethanol (final concentration 20 mM) was added to quench EDC. The activated collagen was separated from the excess reducing agent and the inactivated cross-linker using Ultra-4 Centrifugal Filter Units (100 kDa), and the collagen was rinsed with 3 mL MES buffer (pH 7.0, 50 mM). The activated collagen was mixed with the HBA-PEI-PGE_2_ conjugates and allowed to react overnight with stirring at 4°C, and then was ultrafiltered and washed with sterile H_2_O to remove the unreacted conjugates and cross-linking reagents by using Ultra-4 Centrifugal Filter Units (100 kDa) at 4°C. Collagen was collected in PBS and then freeze-dried to yield the final product (COL-PGE_2_).

#### Characterization of the COL-PGE_2_ matrix

The morphology of the COL, PGE_2_/COL and COL-PGE_2_ matrix was assessed by scanning electron microscopy (SEM; HITACHI X-650, Tokyo, Japan). The chemical structures of the COL, PGE_2_/COL and COL-PGE_2_ matrices were characterized using FT-IR. The FT-IR transmittance spectrum in the wavelength region from 4000 to 400 cm^−1^ was acquired using an FT-IR spectrometer (FTS-6000 spectrometer; Bio-Rad, Hercules, CA). Furthermore, the rheological properties of the COL, PGE_2_/COL and PGE_2_ matrices were measured on a 25-mm parallel-plate rheometer (TA Instruments, New Castle, DE) as previously reported. In summary, the elastic moduli (G′) and the viscous moduli (G″) of the COL, PGE_2_/COL and COL-PGE_2_ matrices at different temperatures from 4°C to 42°C were measured at a frequency of 0.159 Hz. The rheologic properties of the samples were measured within a temperature range of 4°C–42°C with a constant heating rate of 2°C/min. The changes in the elastic (storage) modulus (G′) and the viscous (loss) modulus (G″) were recorded as the change in temperature at a fixed frequency of 1 rad/s. The phase lag (δ) was used to determine the gelation temperature at which the elastic modulus (G′) and the viscous modulus (G″) were equivalent.

#### Biocompatibility of the COL-PGE_2_ matrix

To determine the optimal concentration of the COL-PGE_2_ matrix for pRTEC proliferation, a cell counting kit-8 assay was used to detect cell proliferation of cells cultured in 96-well plates coated with no coating, collagen-coated, PGE_2_/COL coated or COL-PGE_2_ matrix-coated 96-well plates for 48 h and 72 h. To investigate the biocompatibility of the COL-PGE_2_ matrix, 96-well plates were coated with collagen, PGE_2_/COL or COL-PGE_2_ matrix and 1×10^4^ pRTECs were added per well. Cell viability was assessed using Trypan blue cell counting with Trypan blue. Cell viability was calculated and normalized according to the initial viability of the culture.

#### Measurement of PGE_2_ release

For the *in vitro* PGE_2_ release assay, 200 μL of the COL/PGE_2_ matrix and the COL-PGE_2_ matrix were deposited at the bottom of a 1.5 mL test tube, incubated at 37° C for 15 min, and covered with a layer of 1× PBS buffer. Two hundred microliters of collagen that was cross-linked with PGE_2_ or was not cross-linked was deposited at the bottom of a 1.5 mL microcentrifuge tube. After incubation at 37°C for 15 min, 500 μl of 1× PBS was carefully layered on top of collagen. The mixture was then incubated at 37°C for up to 16 days. One hundred microliters of solution were removed from the PBS layer at different time points and replaced with 100 μl of fresh 1× PBS. PGE_2_ was measured by a chemiluminescence enzyme immunoassay (K051-H1, Assay Design, Inc., Ann Arbor, MI).

For the *in vivo* PGE_2_ release assay, 75 μl of COL, PGE_2_/COL, and COL-PGE_2_ matrix were transplanted into the renal capsule of C57BL/6 mice (7-8 weeks old, weight 25-30 g) after AKI. Mice were anesthetized with avertin at the indicated time points (days 1, 2, 3, 7, and 14) by intraperitoneal injection. The kidney was then explanted by removing the kidney capsule and matrix, snap frozen in liquid nitrogen, and stored at −80°C until needed. To prepare kidney homogenates, tissue samples were weighed and homogenized in 50 mM Tris buffer, pH 7.5, containing antioxidant butylated hydroxytoluene (10 μM) and the cyclooxygenase inhibitor indomethacin (10μg/mL) to block *ex vivo* arachidonic acid autooxidation and prostaglandin formation and kept on ice. The homogenates were vigorously vortexed and incubated for 5 min on ice before centrifugation at 14,000 rpm for 45 min. The supernatants were collected and stored at −80°C until needed. PGE_2_ was measured by a chemiluminescence enzyme immunoassay (K051-H1, Assay Design, Inc., Ann Arbor, MI).

#### Cell apoptosis assay

To test the cytoprotective effects of the COL-PGE_2_ matrix, 1×10^6^ pRTECs were seeded in 6-well plates coated with non-coated COL, PGE_2_/COL, or COL-PGE_2_ matrix or cultured for 24 h. Cells were then exposed to 150 μM H_2_O_2_ for 6 h. Cells in medium without H_2_O_2_ served as a control. After staining with an Apoptosis Hoechst staining kit (C0003, Beyotime Biotechnology, China) for 10 min, Trizol (Invitrogen, Grand Island, NY) was added for the next step of RNA extraction. After Hoechst staining, the cells were washed with PBS to remove the remaining Hoechst 33342 and observed under a fluorescence microscope (Nikon). Apoptotic cells stained with Hoechst 33342 dye were counted, as they were distinguished by bright blue nuclear staining. The mean fluorescence intensity of the images in seven random fields of view was quantified by ImageJ software (NIH, Bethesda, Maryland).

#### Knockdown of Yap in primary renal tubular epithelial cells

The shRNA (short hairpin RNA) sequences targeting Yap were individually inserted into the PLKO.1 construct. As a negative control, the nonspecific control shRNA (sh Ctrl) was inserted into the construct. Cloning was confirmed by DNA sequencing. To generate lentivirus, the shRNA construct was transfected into HEK-293T cells along with the lentivirus package constructs, pMD2.G and PAX, and allowed to incubate for 16 h. The cells were then transferred to the complete medium and cultured for an additional 24 h. The medium was harvested and concentrated. For infection, virus was added to pRTECs in polybrene (8 μg/mL)-containing medium for 24 h. Subsequently, the medium was removed and replaced with a complete medium containing 1 μg/mL puromycin for 3 days. The expressions of the Yap protein in stable cells were verified by Western blot assays and immunofluorescence staining.

#### Intravital two-photon imaging in mice

An AIW was implanted in the abdomen of the mouse, fixed with an adapter, and maintained under anesthesia by injecting avertin. The objective lens was 25× and immersed in water on the AIW window in the abdomen of the mouse. Two-photon excitation was performed at a wavelength of 835 nm (10% laser transmissivity), and emission was collected at 495–540 nm (EGFP) and 575–630 nm (td Tomato). Scanning was advanced with Z steps of 5 μm and zoom under the 25× objective lens (580 μm × 580 μm single scanning area) and an 800 × 800-pixel size.

#### Renal function analysis

For the assessment of renal function, at different time points after injury, blood samples were taken and serum was collected for the assessment of BUN and creatinine using the Colorimetric Detection Kit of Urea Nitrogen (BUN) Colorimetric Detection Kit (C013-1, Nanjing Jiancheng Institute of Bioengineering) and the Creatinine Assay Kit (C011, Nanjing Jiancheng Institute of Bioengineering).

#### Histological analysis of renal tissues

At the indicated time points, the animals were sacrificed to harvest kidney samples. For paraffin sections, kidney samples were fixed with 4% paraformaldehyde, dehydrated with ethyl alcohol, hyalinized with xylene, and eventually embedded in paraffin (Leica Microsystems, Wetzlar, Germany). For cryosection, kidney samples were fixed with 4% paraformaldehyde, dehydrated with a 30% sucrose solution and embedded in Optimal Cutting Temperature (OCT) compound (Sakura Finetek, Tokyo, Japan). All samples were cut into a series of sections with a thickness of 5 μm. Hematoxylin and eosin (H&E) staining and Masson trichrome staining were performed on paraffin sections according to a standard protocol, while frozen sections were used for immunofluorescent staining. Quantitative immunostaining analysis was performed with ImageJ software. Histological examinations were performed by three blinded renal pathologists. Histological changes caused by tubular necrosis were quantified by calculating the percent of tubules that exhibited cast formation, loss of the brush border, tubular cell necrosis as follows: 0, none; 1, 10%; 2, 11% to 25%; 3, 26% to 45%; 4, 46% to 75%; and 5, >76%. At least 10 nonoverlapping visual fields (×200) were reviewed for each kidney in the HE stained specimens. Analyzes were performed blindly to treatment assignments in all experiments.

#### Western blot analysis

Cells were lysed on ice in radioimmunoprecipitation assay (RIPA) buffer (Solarbio, Shanghai, China), and total protein was quantified using a BCA Protein Assay Kit (Thermo Scientific). For tissue protein analysis, renal capsules and matrix were removed from mouse kidney tissue, cut into a 1.5 mL EP tube supplemented with a proteinase inhibitor cocktail (Sigma-Aldrich) and transferred to a homogenizer. The tissue homogenates were lysed on ice with RIPA buffer for 30 min. Total proteins were diluted in 4× SDS-PAGE loading buffer and boiled for 5 min. The harvested proteins were separated by 10% SDS-PAGE and transferred to polyvinylidene fluoride membranes (PVDF; Millipore, Darmstadt, Germany). After blocking with 5% non-fat milk for 2 h, the PVDF membranes were incubated with primary antibodies overnight at 4°C and then for 2 h at room temperature with secondary antibodies. Primary antibodies included β-tubulin (1:1000, 10068-1-AP, Proteintech Group, Wuhan, China), YAP (sc-271134, 1:500, Santa Cruz Biotechnology, CA), p-YAP (13008, 1:1000, Cell Signaling Technology, MA, USA), Arge (ab180722, 1:1000, Abcam), survivin (sc-17779, 1:500, Santa Cruz Biotechnology, CA), Sox9 (ab185966, 1:1000, Abcam), p-Lats1 (8654, 1:1000, Cell Signaling Technology, MA, USA); β-tubulin was used as an internal control.

#### Quantitative real-time PCR

Total RNA was isolated from cells or tissues using TRIzol reagent (Invitrogen, Grand Island, NY) and purified using RNeasy columns (Qiagen, Chatsworth, CA). First-strand cDNA was synthesized by reverse transcriptase (TransGen Biotech, China) using oligodT primers. Subsequently, the Hieff qPCR SYBR Green Master Mix Kit (Yeasen, China) was used to quantify mRNA expression levels in 20 μl reaction volumes. Real-time PCR analysis was performed on the Opticon® System (Bio-Rad, Hercules, CA). The 2^−ΔΔCt^ method was used to analyze relative gene expression. The sequences of the primers are listed in the Key resources table.

### Quantification and statistical analysis

All presented results were obtained from at least three independent experiments for each condition. Statistical analyzes were performed using GraphPad Prism software (GraphPad Software Inc., San Diego, CA). One-way repeated measures ANOVA with Tukey post hoc tests, two-way MANOVA with Tukey post hoc tests, and two-way repeated measures ANOVA with Sidak’s post hoc test were used. Differences were considered significant at a *P* value < 0.05. The analyzes were performed blindly in all experiments.

### Additional resources

None.

## Data Availability

Any additional information required to reanalyze the data reported in this paper is available from the lead contact upon request. No original code was produced in this study.
