# Supplementary material for: Renal subcapsular delivery of PGE2 promotes kidney repair by activating endogenous Sox9+ stem cells
Source: iScience. 2021 Oct 8;24(11):103243. doi: 10.1016/j.isci.2021.103243 (PMC8554536; doi:10.1016/j.isci.2021.103243)
Supplement: Document S1. Figures S1–S — 10 [file mmc1.pdf]

iScience, Volume *24*

Supplemental information

Renal subcapsular delivery of PGE_2 promotes kidney repair by activating endogenous $Sox9^+$ stem cells

Shang Chen, Haoyan Huang, Yue Liu, Chen Wang, Xiaoniao Chen, Yuqiao Chang, Yuhao Li, Zhikun Guo, Zhibo Han, Zhong-Chao Han, Qiang Zhao, Xiang-Mei Chen, and Zongjin Li

iScience, Volume ■■

Supplemental information

Renal subcapsular delivery of PGE_2 promotes kidney repair by activating endogenous $Sox9^+$ stem cells

Shang Chen, Haoyan Huang, Yue Liu, Chen Wang, Xiaoniao Chen, Yuqiao Chang, Yuhao Li, Zhikun Guo, Zhibo Han, Zhong-Chao Han, Qiang Zhao, Xiang-Mei Chen, and Zongjin Li

SUPPLEMENTAL INFORMATION

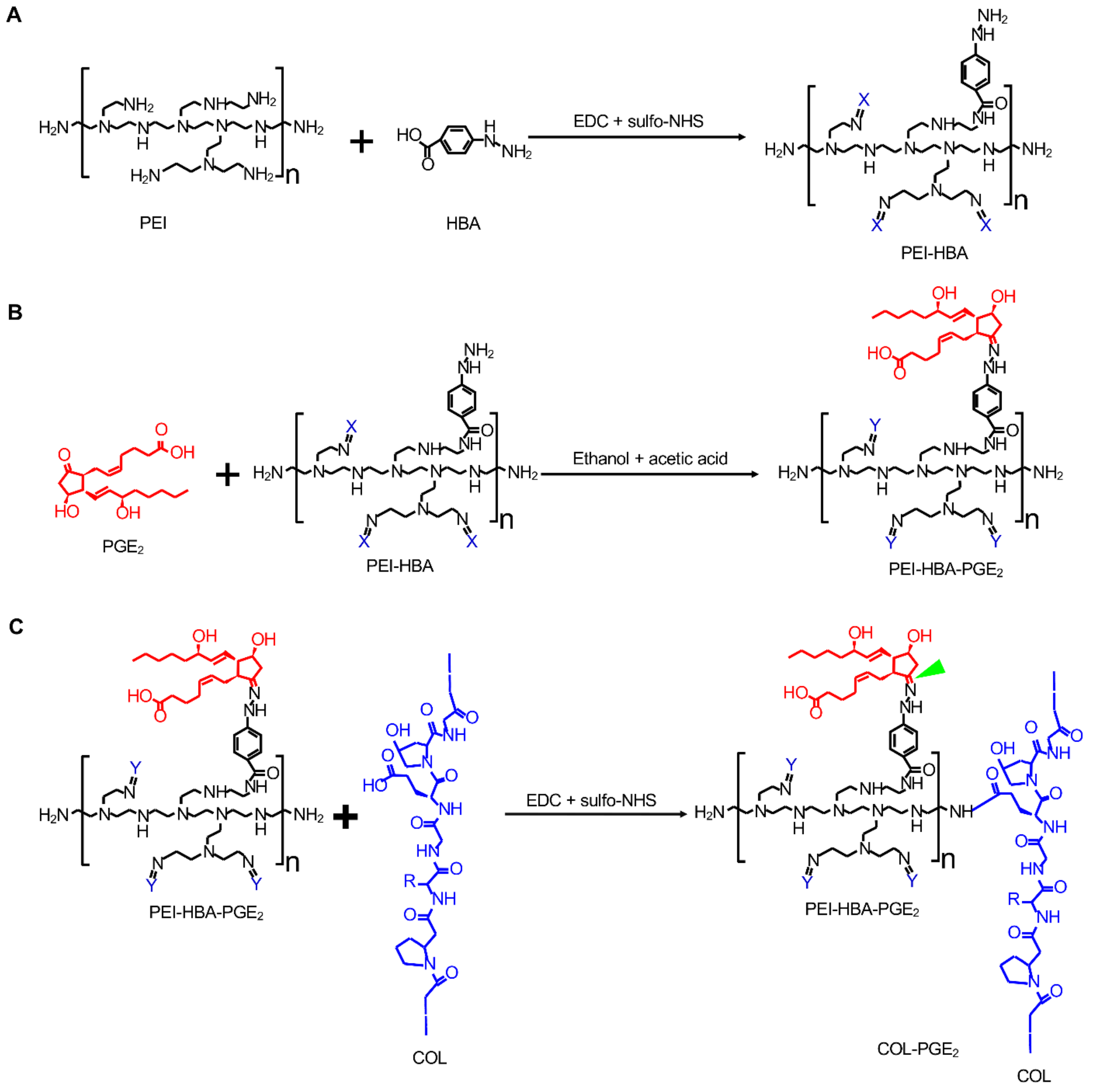

Figure S1. A schematic depicting the method of synthesis of the COL-PGE$_2$ matrix. (**A**) The PEI-HBA conjugates were synthesized by the dehydration condensation reaction between the amine group on PEI and the carboxylic group on HBA. (**B**) The ketone group on PGE$_2$ was reacted with the hydrazine group on PEI-HBA to synthesize the PEI-HBA-PGE$_2$ conjugates through a dehydration reaction. (**C**) The PEI-HBA-PGE$_2$ conjugates and collagen were further crosslinked by using dehydration condensation. Mechanism of PGE$_2$ release: The breakage sites of PGE$_2$ covalently attached (red) to branched PEI-HBA (black) and collagen (blue) are indicated by green arrows. X= hydrogen or HBA; Y = hydrogen, HBA or HBA-PGE$_2$. Abbreviations: HBA, 4-hydrazinobenzoic acid; PEI, polyethyleneimine.

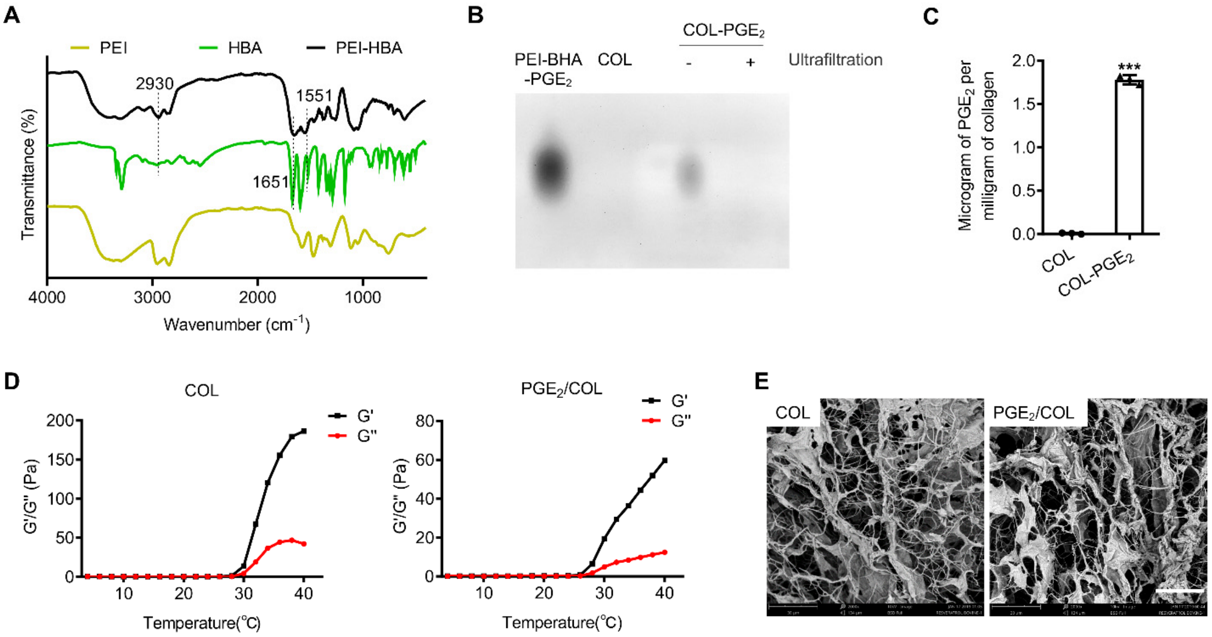

Figure S2. Characterization of collagen and the PGE2/COL matrix. (**A**) FT-IR analysis shows the characteristic peaks of PEI, HBA, and PEI-HBA. (**B**) Tris-borate-EDTA–PAGE was used to detect free PEI-HBA-PGE2. (**C**) Quantification of PGE2 in collagen before and after crosslinking as determined using ELISA. Results are normalized to collagen concentration and expressed as the content of PGE2 per milligram of collagen. The two-tailed *t* test was used for statistical analysis. Data are expressed as mean ± SD; $^{***}P < 0.001$ versus COL. The experiments were carried out in triplicate. (**D**) Evaluation of the rheologic profile of collagen (left) and the PGE2/COL matrix (right) during temperature changes. (**E**) Scanning electron micrograph image that reveals the morphologic structure of lyophilized collagen (left) and the PGE2/COL matrix (right). Scale bars, 30 μm.

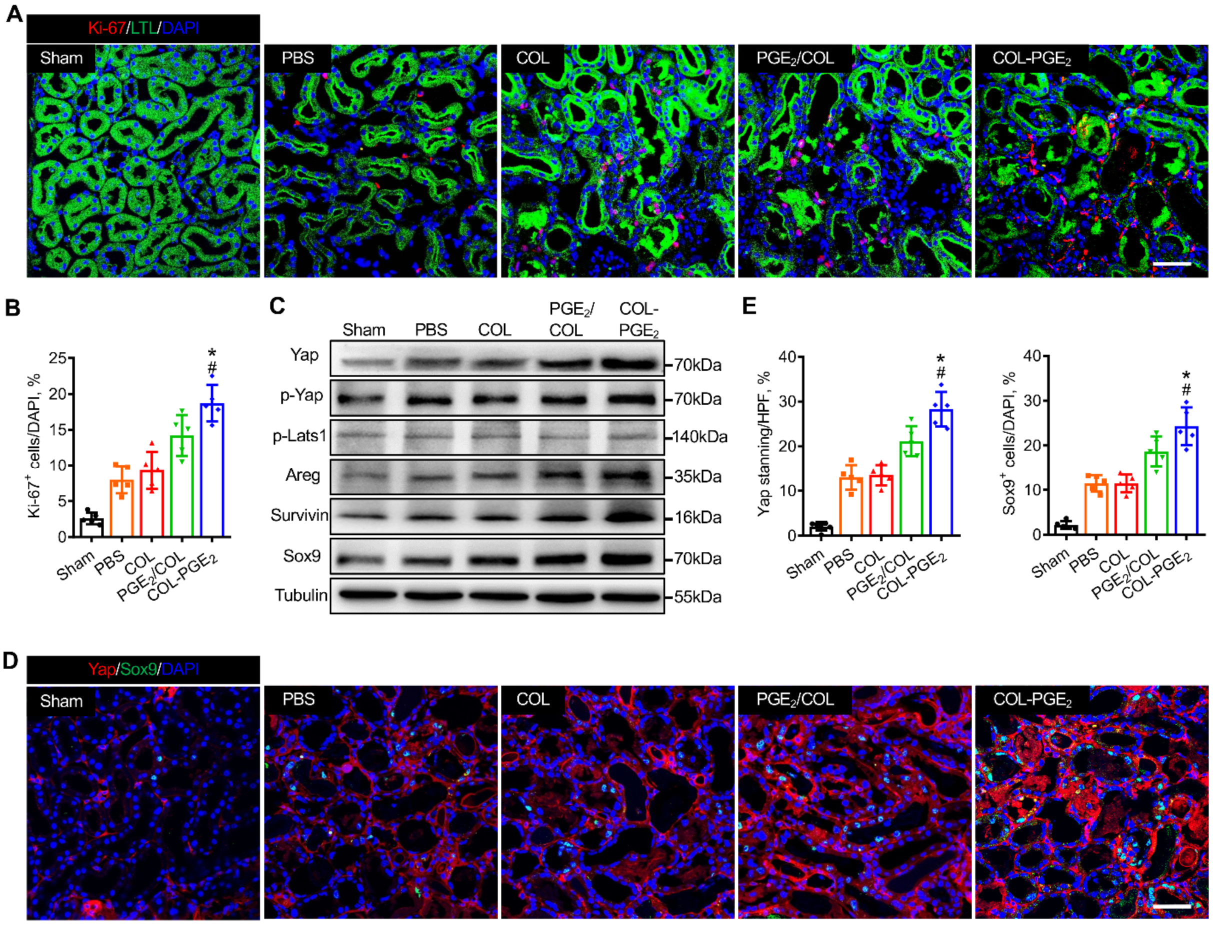

Figure S3. COL-PGE2 promoted the proliferation of proximal tubular epithelial cells and the activation of Sox9 through Yap mediation. (**A**) Representative images show Ki-67 immunostaining (red) detected in proximal tubular epithelial cells (LTL, green) on day 3 after AKI. (**B**) Quantification of Ki-67$^+$ staining in (**A**). (**C**) Immunoblot analysis of the YAP, p-YAP, p-Lats1, Arge, Survivin, and Sox9 protein in the kidney on day 3 of PBS, COL, PGE_2/COL and COL-PGE2. (**D**) Immunostaining analysis of YAP (red) and Sox9 (green) on day 3 after AKI. (**E**) Quantification of YAP$^+$ (left) and Sox9$^+$ (right) staining. HPF, high-power field. Scale bars, 50 μm. One-way repeated measures ANOVA with Tukey post hoc tests (B and E) was used for statistical analysis. Data are expressed as mean ± SD; n=5, $^*P < 0.05$ versus COL; $^\#P < 0.05$, versus PGE_2/COL.

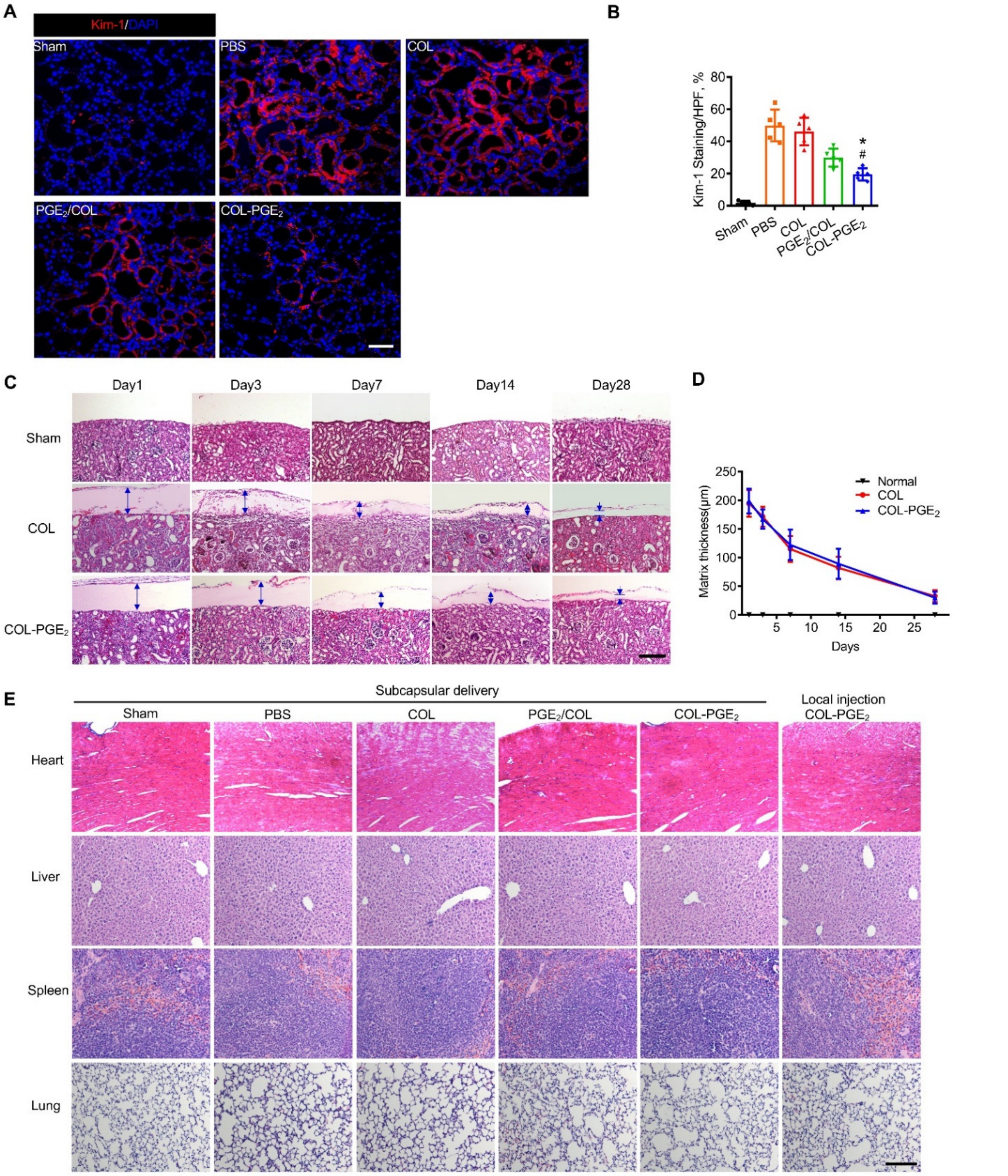

Figure S4. The COL-PGE2 matrix attenuates kidney injury without organ toxicity. (**A**) Histological analysis of kidney injury by anti-Kim-1 immunostaining (red) on day 3 post AKI. Scale bars, 50 μm. (**B**) Quantification of Kim-1^{+} staining. (**C**) Analysis of the matrix degradation profile by measuring the thickness of the matrix under the kidney capsules on day 1, 3, 7, 14, 28 post AKI. Scale bars, 200 μm. (**D**) Quantification of the thickness of the matrix. (**E**) H&E staining did not show histological changes in the heart, liver, spleen and lung. One-way repeated measures ANOVA with a Tukey post hoc test (**B**) and Two-way multivariate analysis of variance (two-way MANOVA) with Tukey post hoc test (D) were used for statistical analysis. Data are expressed as mean ± SD; n=5, **P* < 0.05 versus COL; $^{\#}P$ < 0.05, versus PGE_2/COL.

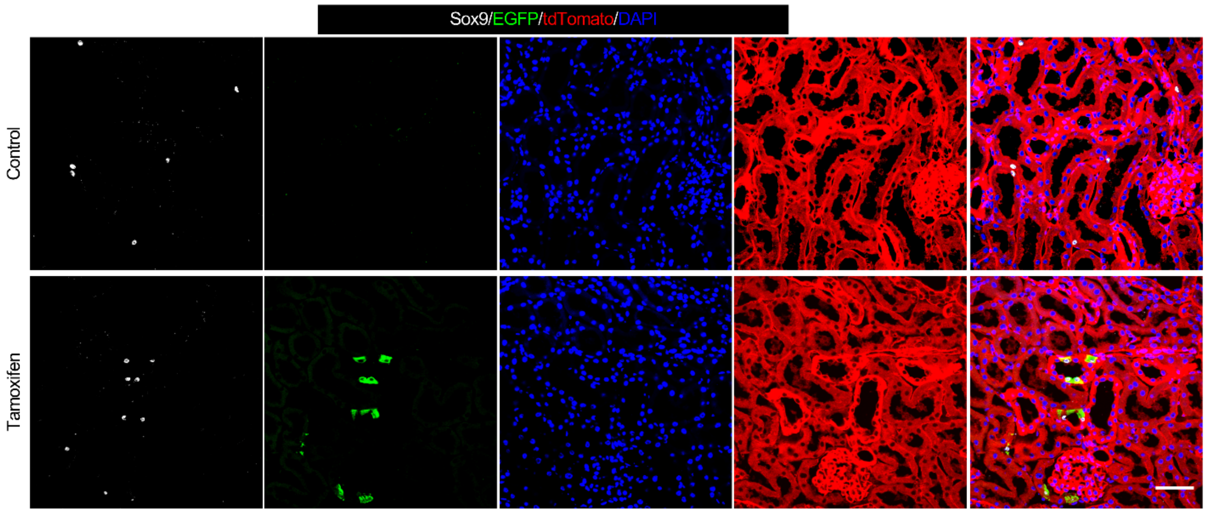

Figure S5. Validation of the Sox9-Cre^{ERT2}; R26^{mTmG} mouse genetic model
To verify the mouse genetic model, tamoxifen (corn oil was used as a control) was injected three times in succession before AKI, and Sox9^{+} cells and EGFP fluorescence colocalization in the kidney were detected by anti-Sox9 immunostaining.

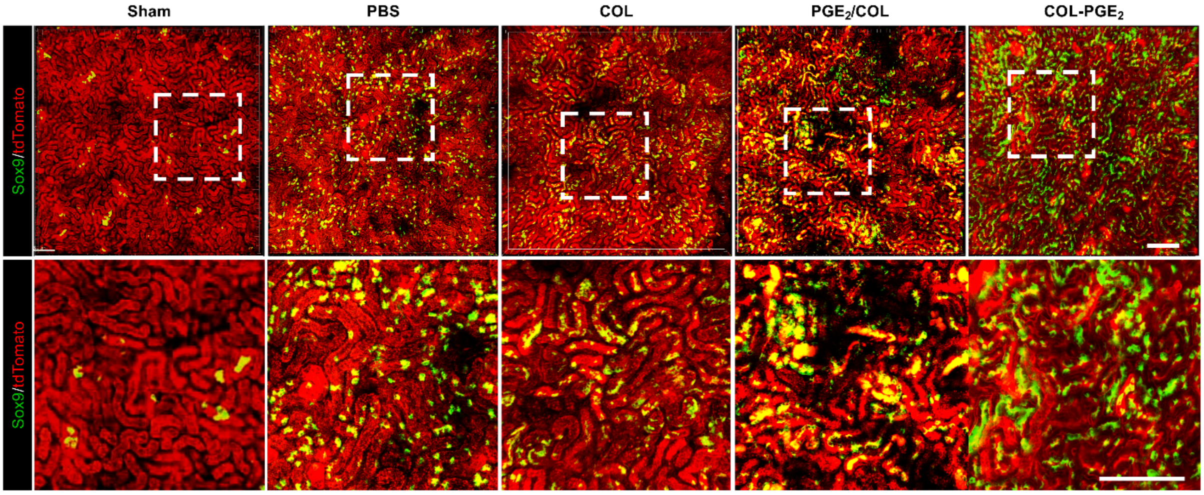

Figure S6. Two-photon living imaging of $Sox9^+$ cell expansion following PGE_2 treatment. Representative images of two-photon intravital tracing showed that $Sox9^+$ cell-derived cells were abundantly expanded by the administration of COL-PGE_2 matrix on day 7 after AKI. Scale bar, 200 μm. See also Video S1-4.

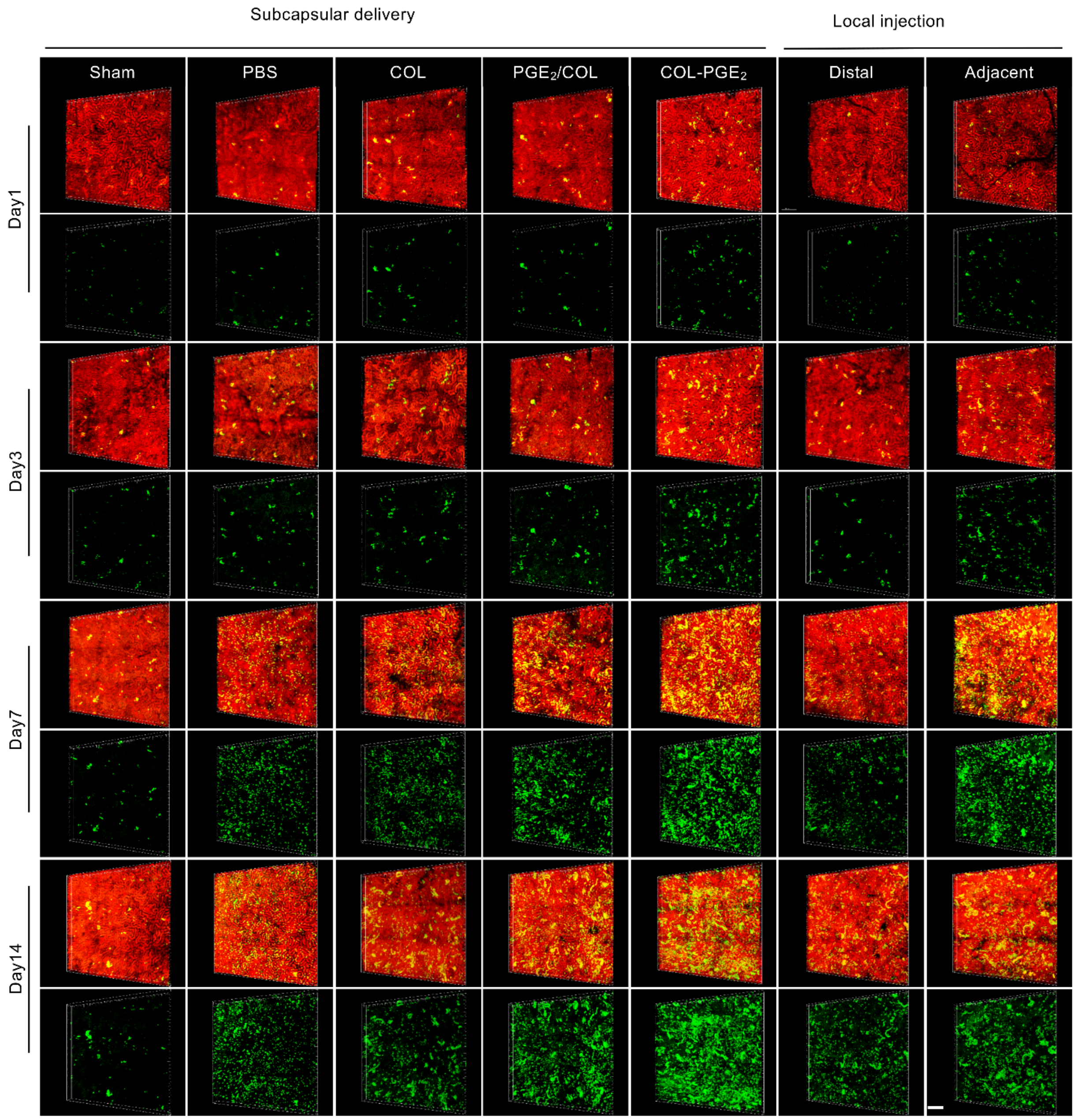

Figure S7. The COL-PGE2 matrix promoted the formation of functional renal tubules in descendants of Sox9[+] cells. Representative images of two-photon intravital microscopy showed Sox9+ derived cells with the administration of PBS, COL, PGE2/COL and COL-PGE2 matrix at different time points after AKI. Scale bar, 200 μm.

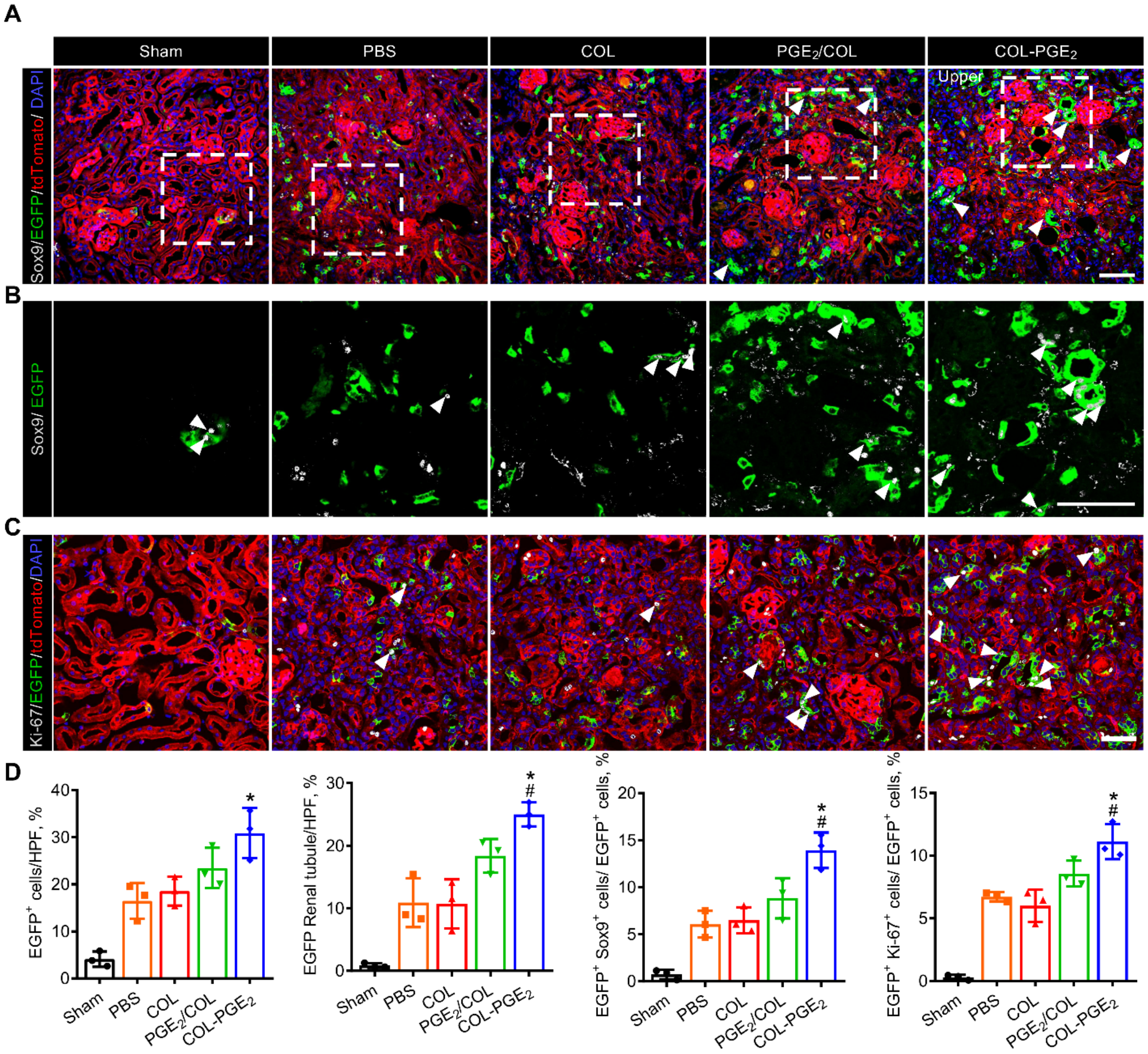

Figure S8. The COL-PGE2 matrix results in increased activation of Sox9 expression and Sox9$^+$ cell proliferation (**A**) Confocal images and (**B**) local zoomed-in images for colocalization analysis of anti-Sox9 immunostaining (gray) and Sox9-Cre^{ERT2}-activated EGFP fluorescence in kidneys with the administration of the PBS, COL, PGE_2/COL and COL-PGE_2 matrix on day 14 post AKI. White arrowheads highlight Sox9$^-$ EGFP-labeled cells, and white arrowheads highlight Sox9$^+$/EGFP-colabeled cells. Scale bar, 50 μm. (**C**) Confocal images for the colocalization analysis of anti-Ki-67 immunostaining (gray) and Sox9-Cre^{ERT2}-activated EGFP fluorescence in kidneys with the administration of PBS, COL, PGE_2/COL and COL-PGE_2 matrix on day 14 after injury. White arrowheads highlight Ki-67$^+$/EGFP colabeled cells. Scale bar, 50 μm. (**D**) Quantification of EGFP+ cells, EGFP renal tubules, Sox9$^+$/EGFP colabeled cells and Ki-67$^+$/EGFP colabeled cells in the kidneys of mice on day 14 after delivery of the COL-PGE_2 matrix post AKI. One-way repeated measures ANOVA with a Tukey post hoc tests (D) were used for statistical analysis. Data are expressed as mean ± SD; n=3, $^*P < 0.05$ versus COL; $^\#P < 0.05$, versus PGE_2/COL.

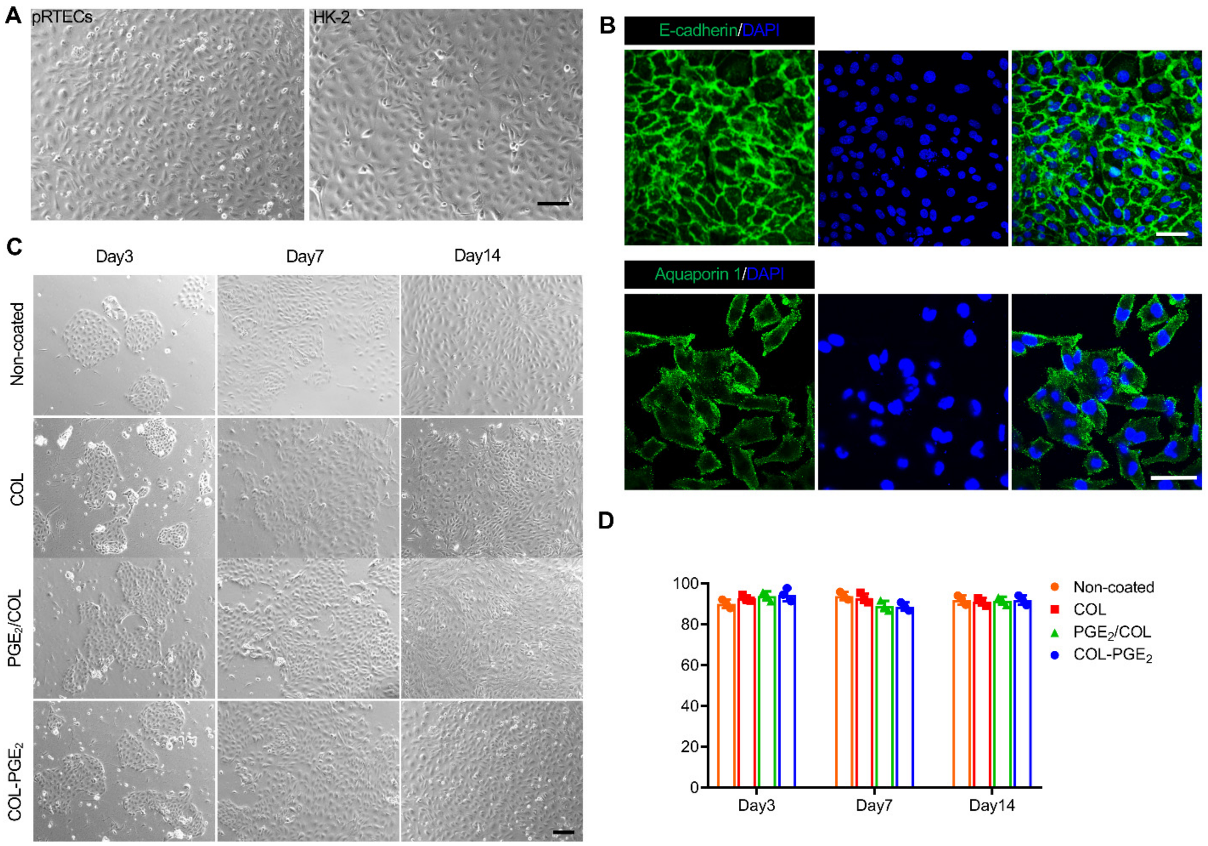

Figure S9. Renal tubular epithelial cell morphology and matrix biocompatibility. (**A**) Comparison of the morphology between isolated primary renal tubular epithelial cells (pRTEC) and HK-2 human renal proximal tubule cells. Scale bar, 100 µm. (**B**) Immunofluorescence staining with antibodies against E-cadherin (top) and Aquaporin-1 (bottom) shows the isolated pRTECs. Scale bar, 50 µm. (**C**) The morphology and colony formation capacity of pRTEC cultured on noncoated or COL, PGE_2/COL or COL-PGE_2 matrix for 3, 7, and 14 days. Scale bar, 100 µm. (**D**) Quantification of cell viability by trypan blue staining and calculation of the percentage of live cells cultured on noncoated or COL, PGE_2/COL or COL-PGE_2 matrix-coated plates for 3, 7, and 14 days. Two-way MANOVA with Tukey post hoc test was used for statistical analysis. Data are expressed as mean ± SD; All data are representative of three independent experiments and are shown as mean ± SD.

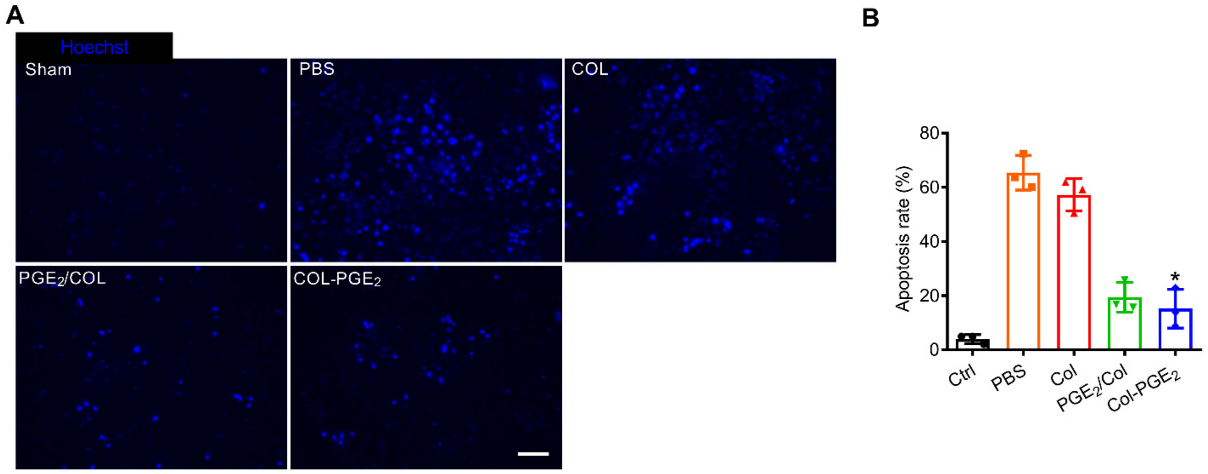

Figure S10. COL-PGE$_2$ matrix exerts an anti-apoptosis effect *in vitro*. (**A**) Hoechst 33342 staining showed the survival of pRTECs cultured on noncoated, PBS, COL, PGE$_2$/COL or COL-PGE$_2$-coated plates upon exposure to 150 μM H_2O_2 for 6 h. Scale bar, 100 μm. (**B**) Quantification of the apoptosis index of pRTECs under H_2O_2-induced stress. One-way repeated measures ANOVA with a Tukey post hoc test (B) was used for statistical analysis. Data are expressed as mean ± SD, n=3, *P < 0.05 versus COL.
